# Application of phage surface display for the identification of Eu^3+^-binding peptides

**DOI:** 10.3389/fbioe.2025.1508018

**Published:** 2025-02-20

**Authors:** Gerda Techert, Björn Drobot, Robert Braun, Christoph Bloss, Nora Schönberger, Sabine Matys, Katrin Pollmann, Franziska L. Lederer

**Affiliations:** ^1^ Biotechnology Division, Helmholtz-Zentrum Dresden-Rossendorf, Helmholtz Institute Freiberg for Resource Technology, Dresden, Germany; ^2^ Department of Biogeochemistry, Helmholtz-Zentrum Dresden-Rossendorf, Helmholtz Institute Freiberg for Resource Technology, Dresden, Germany

**Keywords:** europium, REE recovery, phage surface display, peptides, next generation sequencing

## Abstract

Europium as one of the rare earth elements (REE) has outstanding properties in terms of its application for high-tech and renewable energy products. The high supply risk of REE, coupled with their low recovery rates from secondary sources, necessitates innovative recycling approaches. We introduce a phage display-based peptide biosorbent recycling technology that offers a cost-effective and environmentally friendly solution for recovering metal ions, supporting circular economy goals. In this study, we used phage surface display to screen for peptides with high affinity for europium (III) ions (Eu^3+^). Performing several independent biopanning experiments with the Ph.D.-12 Phage Display Peptide Library and different elution methods as well as combining them with next-generation sequencing, we identified eight peptides with moderate to good affinities for Eu^3+^ ions, verified by time-resolved laser fluorescence spectroscopy. The peptides EALTVNIKREME as well as DVHHVDGNDLQPFEGGGS and DSIHSDVTKDGRYPVEGGGS, the latter are variants of enriched dodecamers, proved to be the best candidates for future biosorption and selectivity studies. This study underscores the potential of phage surface display for peptide-based REE recovery, laying the foundation for selective recycling technologies from secondary raw materials.

## 1 Introduction

Rare earth elements (REE), especially lanthanides (Ln), have become indispensable in times of modern technology due to their special properties. They are not only used for high-tech and green-tech products such as catalysts, screens, metal alloys, fuel cells, permanent magnets and compact fluorescent lamps (Schüler et al., 2011; Adler and Müller, 2014), but are also applied in medicine and agriculture due to their diverse biological effects. For instance, they are used in cancer therapy (Fricker, 2006; Lever et al., 2004) and in the treatment of bone diseases like osteoporosis (Barta et al., 2007) or serve for diagnostic purposes such as the Gd^3+^-based contrast agents in magnetic resonance imaging (Merbach and Tóth, 2001). In Chinese agriculture, Ln and other REE have been used for a long time to increase both plant and animal production. To stimulate the growth of plant and animal organisms, REE salt mixtures including the elements scandium, yttrium and the Ln from lanthanum to lutetium have been added to fertilisers or animal feed (Rambeck et al., 1999; Zohravi, 2006; Ni, 2002; Hu et al., 2004).

China holds the monopoly in REE mining and production, dominating the REE prices and accessibility. The significantly increased demand for REE over the last two decades and the resulting increase in production, as well as the central supplier role of China since the beginning of the 21st century, raised political and economic questions regarding global supply (van Gosen et al., 2017). Published reports by the U.S. Department of Energy in 2011 (U.S. Department of Energy, 2011), IZT/ adelphi in 2011 (Erdmann et al., 2011) and the European Commission in 2017 (Kommission, 2017), for example, point to the high supply risk of REEs, giving them greater attention and appreciation for their strategic importance. This new awareness of the relevance of REE together with climate and environmental protection policies, considering that mining and extraction of REE is associated with enormous environmental burdens, resulted in the search for alternative approaches such as the recycling of REE-containing end-of-life (EOL) products or industrial and mining wastewater. REE recovery through recycling and circular economy offers an immense potential. This was the conclusion reached by researchers at Yale U.S. University, estimating that the amount of rare earth oxides (REO) in products in use worldwide was about 440,000 metric tons in 2007. This represented four times the amount of REE mined in the same year (Du and Graedel, 2011). However, separation processes for Ln are challenging in terms of costs and efficiency due to low concentrations in EOL products and wastewaters and their similar chemical and physical properties. The current recycling rates of REE are therefore less than one percent (Graedel et al., 2011). For this reason, research has been increasingly focusing on new recycling methods that enable cost-effective and environmentally friendly separation of REE from electronic scrap and wastewater. These approaches help reduce the strain on limited primary raw material sources and achieve greater independence from insecure supply chains, which are often tied to mining activities concentrated in a few regions worldwide.

Furthermore, the intensive mining of REEs in producing countries, coupled with their increasing use in everyday applications, has resulted in their accumulation in the environment (Gonzalez et al., 2014) and the human body, posing environmental and health risks. Despite being used in agriculture and animal feed, some concerns have been raised regarding the potential chemo- (Pagano et al., 2015a; Pagano et al., 2015b) and cytotoxic (Wang et al., 2016; Gao et al., 2017) effects of Ln, with contradictory data found in literature. The toxicity of Ln appears to be concentration-dependent (Pagano et al., 2015b) due to the similarity in coordination chemistry between Ln ions (Ln^3+^) and calcium ions (Ca^2+^), which can impair calcium-mediated biological functions. Calmodulin (CaM), a key molecule in calcium-dependent signal transduction pathways in plants and animals, is considered a potential target of Ln (Hu et al., 2004). Since Ln^3+^ ions are known to have a high affinity for CaM (Drobot et al., 2019; Bruno et al., 1992; Horrocks and Tingey, 1988; Bertini et al., 2003; Wang et al., 1982), they may bind to these holoproteins, blocking signal transduction pathways or increasing the concentration of these ions in healthy cells, potentially leading to accumulation in human organs (Zaichick et al., 2011; Wei et al., 2013). Additionally, some radiolanthanides have been approved for cancer radiotherapy (Novartis Pharmaceuticals Corporation, 2024), highlighting their biomedical relevance. The mentioned health risks underscore the need for specific biosorbents for Ln detoxification.

In addition to CaM, other proteins and peptides have been discovered and developed for Ln^3+^ binding, as reviewed by Ye et al. (2024). For instance, lanmodulin (LanM) was found to bind Ln^3+^ in a highly selective manner. It originates from different strains of methylotrophic bacteria, such as *Methylorubrum extorquens* AM1 and *Beijerinckiaceae bacterium* RH AL1. Like CaM, LanM possesses four metal-binding EF-hand motifs (Cotruvo et al., 2018). It is able to bind picomolar concentrations of all Ln^3+^ as well as Y^3+^, and undergoes a strong conformational change from a largely disordered state to a compact, ordered state, which is contrary to other EF-hand-containing proteins. However, it forms a bond with Ca^2+^ only at near millimolar concentrations. Recently, a new natural Ln-binding protein was revealed, Lanpepsy (LanP), which is the first of the PepSY family discovered to selectively bind Ln^3+^ over Ca^2+^ (Hemmann et al., 2023). Furthermore, the introduction of aspartate/glutamate residues into *de novo* designed coiled-coil or triose-phosphate isomerase (TIM) barrels and ferredoxin and the resulting affinity for terbium has also been described (Webster and Peacock, 2021; Caldwell et al., 2020). Although all these described biomolecules bind Ln^3+^ with high affinity, they are not described to discriminate between the elements, making selective recovery difficult. For example, a rational designed, CaM-derived Ln-binding tag (LBT) has been reported to exhibit a >60-fold lower affinity for La^3+^ than for Yb^3+^ (Hatanaka et al., 2020). However, no evidence of selective recovery has been demonstrated for this tag, and further studies are needed. Both the recovery of a particular Ln from minerals, EOL products, or wastewaters, as well as its effective and safe removal from the human organism, require the use of biosorbents that have not only high affinity but also high selectivity for the corresponding Ln.

Peptides hold the potential to act as highly selective Ln^3+^ ligands, which can be further immobilized on a suitable carrier material to act as biosorbents for Ln recovery and detoxification. A similar concept has already been demonstrated for other valuable industrial metals like gallium (Schönberger et al., 2019), nickel and cobalt (Matys et al., 2020) or lead (Nian et al., 2010). Peptides offer several advantages as biomolecules in this context. Their selectivity is determined by their amino acid composition and secondary structure, which can be easily modified or functionalized to suit specific applications. Unlike proteins, peptides are typically more stable under variations in temperature and pH, a property particularly valuable in industrial wastewater. Their simpler structure ensures that their functionality remains largely unaffected by these fluctuations, making them more reliable for use in toxic environments. Peptides can also be produced in large quantities at relatively low cost, either through solid-phase peptide synthesis or heterologous expression, depending on their size and complexity. Furthermore, their structure allows for the incorporation of functional groups or non-natural amino acids, facilitating immobilization onto applicable matrices, such as polymers, for industrial use. Due to their simpler structure compared to proteins, site-directed functionalization is also easier to achieve in peptides, enhancing their versatility for specific applications. A promising approach for the identification of artificial Ln-binding biomolecules with high affinity for specific Ln^3+^, such as Eu^3+^, is the phage surface display (PSD). PSD, developed by George Smith in 1985, is a powerful method for selecting and identifying high-affinity biomolecules (Smith, 1985). Phage libraries present an enormous molecular diversity (up to 10^12^) of natural or random peptides, protein domains or complete proteins on the surface of bacteriophages, where each displayed molecule sequence can be identified via the coding DNA of the phage, effectively tagging the biomolecules by their DNA (Kay and Hoess, 1996). Screening of such a randomized phage peptide library ultimately allows the identification of high-affinity peptide ligands for a target material of interest (Smith, 1985). The most widely used method for selecting desired phage clones is the *in vitro* binding incubation, often referred to as biopanning. Other methods, such as enzymatic selection or *in vivo* binding, have also been reported (Russel et al., 2004). In the biopanning process, the initial phage peptide library is incubated with a target that is either previously immobilized to a matrix (for ions, proteins) (Nian et al., 2010) or used directly (for insoluble targets) (Curtis et al., 2017; Lederer et al., 2017; Lederer et al., 2019). Target-selective peptides are enriched through multiple biopanning selection rounds and identified via Sanger or next-generation sequencing (NGS).

In this study, we focused on the identification of Eu^3+^-binding peptides using PSD. Using the deep sequencing tool NGS and by performing several independent biopanning experiments with different elution methods, we were able to compare and correlate enriched sequences across various elution fractions. By incorporating parallel or subsequent negative controls (= negative selection), we further identified and reduced target-unspecific sequences and minimized bias towards propagation-favoured peptides. Peptides identified in this way were investigated for their Eu^3+^ complexation characteristics by Time-Resolved Laser-induced Fluorescence Spectroscopy (TRLFS) experiments. This study is the first to select Eu^3+^-binding peptides using PSD. However, a high selectivity of the peptides is particularly interesting for future applications in the field of REE separation. In future studies, promising peptides will be tested and optimized for a selective recovery of Ln^3+^.

## 2 Materials and methods

### 2.1 Immobilization of Eu^3+^

One of the most critical points in PSD is the presentation of the target material to the bacteriophages. For affinity selection, the Eu^3+^ ions, intended to serve as target elements, had to be immobilized on nitrilotriacetic acid (NTA), which in turn is coupled to agarose beads (PureCube 100 NTA Agarose, Cube Biotech, Monheim, Germany), forming a stable complex with Eu^3+^. To remove the ethanol, NTA-agarose beads were washed six times with Millipore water and sedimented by centrifugation. The beads were mixed with europium chloride solution (10 mM EuCl_3_, 100 mM KCl, pH 6.5) and incubated overnight at 4°C in an overhead shaker (99 rpm). The next day, Eu-NTA agarose beads were washed 10 times with TBST buffer (TRIS-buffered-saline with Tween 20; 50 mM TRIS-HCl, 150 mM NaCl, 0.1% (v/v) Tween 20, pH 7.5), centrifuged, and refilled to the initial volume (10 mL) with the same buffer and served as target for the first biopanning. To investigate the number of immobilized Eu^3+^ ions, three reference samples were taken at 200 μL each (100 μL settling volume) and the metal ions were eluted with 800 μL 1 M nitric acid (HNO_3_) each for 1 h at 70°C. The Eu^3+^ concentrations of the samples were then determined by inductively coupled plasma mass spectrometry (ICP-MS).

### 2.2 Phage display library system

All biopanning experiments were performed using the commercially available pIII-displayed peptide library Ph.D.-12 (Ph.D.™-12 Phage Display Peptide Library Kit, New England Biolabs GmbH, Frankfurt am Main, Germany (NEB)]. A detailed description of the library can be found in the manufacturer’s instructions.

### 2.3 Preparation of host cells

For the amplification of the M13 phage of the peptide library Ph.D.-12, the bacterial strain *Escherichia coli* K12 ER2738 (F’*proA+B+ lacIq Δ(lacZ)M15 zzf::Tn10(TetR)/fhuA2 glnV Δ(lac-proAB) thi-1 Δ(hsdS-mcrB)5*; NEB) served as host. Host cells were used for both titration and amplification of the infectious phage particles.

### 2.4 Phage titering

After each round of selection, the phage particle concentration (phage titer) in the eluate and amplicon was determined. For that, a fresh *E. coli* culture was first prepared by inoculating LB medium (10 g/L bacto-tryptone, 5 g/L NaCl, 5 g/L yeast extract, pH 7.5) with the host strain and culturing it on a shaker at 37°C to *log*-phase with an optical density (λ = 600 nm; OD_600_) of 0.5. Appropriate dilution series of each of the phage fractions were prepared in TBST buffer containing 0.5% (v/v) Tween 20 and 10 μL of each dilution were incubated with 200 μL fresh cells each for 5 min at room temperature. The infected *E. coli* cells (15 µL) were mixed with 200 μL of liquid top agarose (LB medium containing 7 g/L agarose) and placed in one well each of a pre-warmed LB/IPTG/X-Gal 24-well plate (LB medium containing 15 g/L agar, 0.05 mg/mL IPTG (isopropyl-β-D-thiogalactopyranoside) and 0.04 mg/mL X-Gal (5-bromo-4-chloro-3-indoxyl-β-D-galactopyranoside). The plate was incubated at 37°C overnight. By counting the blue appearing plaques, the phage titer could be determined as plaque forming units (pfu).

### 2.5 Phage amplification and purification

Depending on the elution protocol, slightly different phage amplification methods were used. For the propagation of phage particles, the entire eluate (minus 10 μL for titration) was added to either 30 mL of LB medium (biological elution protocol) or to 30 mL of a freshly grown *E. coli* culture (OD_600_ ∼ 0.5; glycine-HCl elution protocol). Phage-infected host cells were incubated for 4.5 h at 37°C and 210 rpm. To separate the phages secreted into the medium from the cells, the culture was centrifuged twice (10,000 × g, 4°C, 10 min). For precipitation of phage particles, the supernatant was mixed with 1/6 of the volume of PEG/NaCl solution (20% (w/v) polyethylene glycol 8,000, 2.5 M NaCl) and incubated at 4°C overnight. The next day, phages were sedimented by centrifugation (10,000 × g, 4°C, 15 min) and resuspended in 1 mL TBS buffer (pH 7.5). In a subsequent purification step, a second precipitation was performed by adding 1/6 of the volume of PEG/NaCl solution to the suspension again and incubating it on ice for 1 hour. Phages were repeatedly spun down (14,000 × g, 4°C, 15 min) and the phage pellet was carefully resuspended in a final volume of 200 μL TBS buffer. Remaining contaminants as well as denatured residues could be removed by a final 1-min centrifugation. The amplicons were stored at 4°C.

To determine possible amplification advantages of individual sequences via NGS, the naïve phage library was also amplified. Two 1 L flasks, each containing 100 mL terrific broth medium (12 g/L bacto-tryptone, 24 g/L yeast extract, 4 g/L glycerol, 8.5 mM KH_2_PO_4_, 36 mM K_2_HPO_4_), were inoculated with 1 mL *E coli* preculture. The cultures were incubated with vigorous shaking (250 rpm) at 37°C to an OD_600_ of around 2.0. The shaking speed was then reduced for five minutes (100 rpm) to allow pili regeneration. This was followed by infection with approximately 10^12^ pfu to each batch and a 15 min incubation at 37°C with slow shaking. The two cultures were then divided into six pre-warmed 1 L flasks, each containing 300 mL LB medium, and incubated at 37°C for 35 min with vigorous shaking. Tetracycline (18 μg/mL) was added and 7 µL samples were taken for titer determination, for which they were diluted with *E. coli* culture and then plated. The six amplification cultures were incubated overnight at 37°C with vigorous shaking before the phage clones were precipitated as described above, combined from the six batches and stored at 4°C.

### 2.6 Biopanning experiments

**FIGURE 1:**
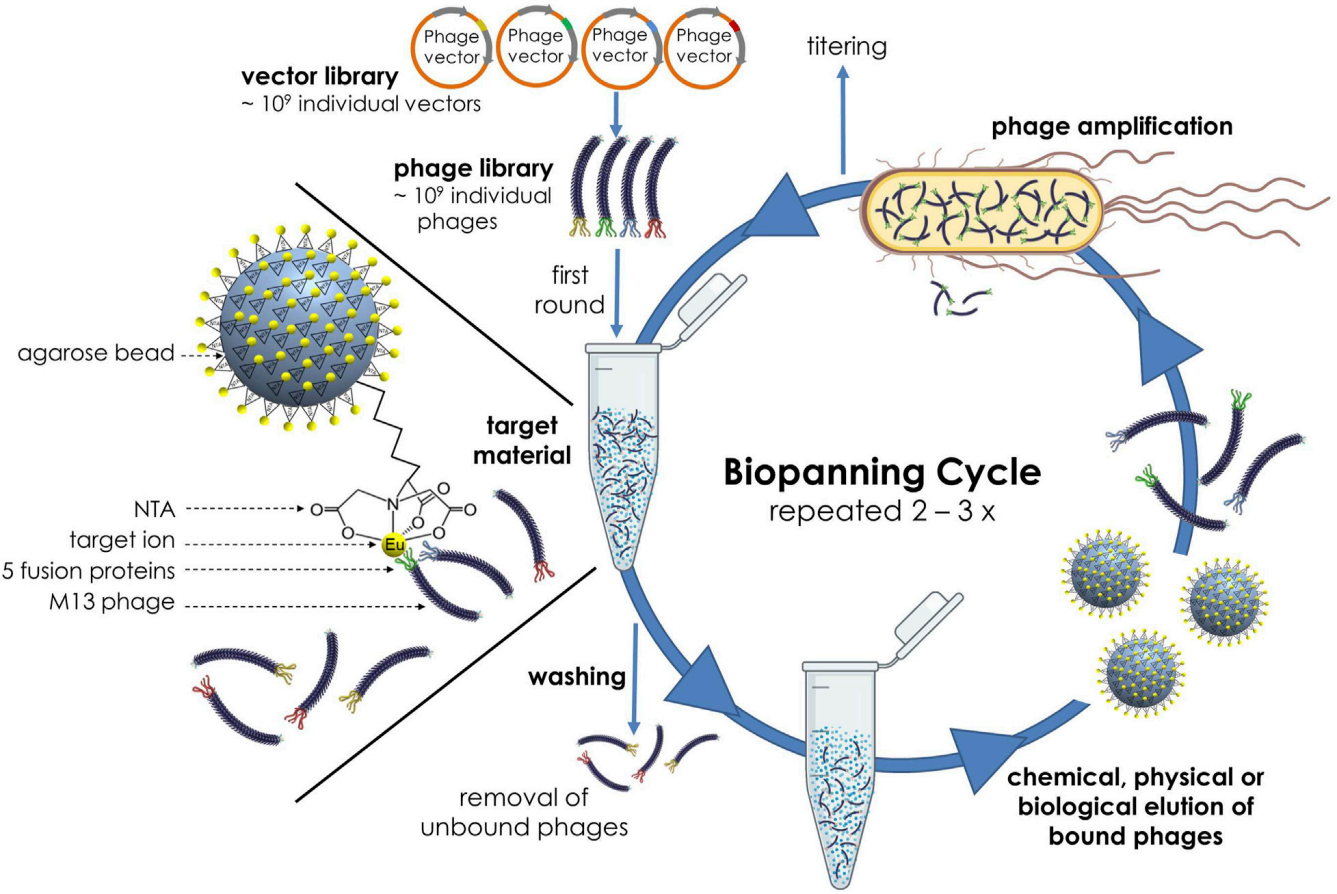
Schematic illustration of the biopanning process with the Ph.D.™-12 Phage Display Peptide Library and Eu^3+^ ions immobilized on NTA agarose beads as target element.

The identification of novel target binding peptides requires for every target a well-defined biopanning protocol. In this biopanning set-up, the naïve Ph.D.-12 peptide library was incubated with Eu^3+^ as target ions, which were previously immobilized to a NTA agarose matrix (see 2.1). While the complexes of bound phages and target element were retained on the solid phase, the unbound phages remaining in solution could be removed by washing several times. For the following elution step of the bound phages from the target material, we used *E. coli* cells for biological elution as well as glycine-HCl or glycine-HCl in combination with ultrasound for chemical or chemical-physical elution in several independent biopanning experiments. In a final step, the eluted phages were amplified in the host organism *E. coli*, titered and used at a concentration of 10^11^ pfu per mL for the next round of selection. Additionally, the stringency was increased by using a higher Tween 20 concentration in the TBST binding and washing buffer with each round. The process was repeated two to three times, until strong-binding bacteriophages have enriched (Figure 1).

#### 2.6.1 Biopanning on Eu^3+^ functionalized carrier material with biological and glycine-HCl elution

One biopanning was performed on Eu-NTA agarose beads without a negative selection step, whereby the phage library is incubated against a material other than the target material (immobilization matrix, other metal-NTA-agarose beads) either before or after the main biopanning with the desired target element. Phages were eluted with *E. coli* cells (biological) or glycine-HCl. For that, 400 μL (≙ 200 µL settling volume) of the prepared Eu-NTA agarose beads were washed two times with TBST buffer (varying Tween 20 concentrations, see end of section) and sedimented by 2–3 min centrifugation. 10 μL of the Ph.D.-12 phage library was mixed with 800 μL of TBST buffer (≙ 10^11^ pfu per mL), of which 10 μL was retained for titration (input), and added to the Eu-NTA agarose beads (total volume of approximately 1 mL). Beads and library were incubated for 1 h in an overhead shaker (99 rpm) and washed 10 times with TBST buffer to remove unbound or only weakly bound phages. Regarding the subsequent elution of phages, it is expected that different elution methods enable the mobilization of different phages from the target. For the biological elution of the strongly bound phage clones from the target material, 200 μL of a freshly grown *E. coli* culture (OD_600_ ∼ 0.5) was added directly to the washed Eu-NTA agarose beads and incubated for five minutes in an overhead shaker (99 rpm). The eluate could be obtained as supernatant by sedimentation of the beads. In contrast, for the chemical elution, 250 µL of 0.2 M glycine-HCl (pH 2.2) was added to the washed Eu-NTA agarose beads and incubated for 10 min in an overhead shaker (99 rpm). After spinning down the beads, the supernatant was neutralized with 44 µL of 1 M TRIS buffer (pH 9.1). The eluates and amplicons were further treated according to the amplification, precipitation and titration described in earlier sections, and the amplified phages (10^11^ pfu per mL) were used as input for the next round of selection. The Tween 20 concentration in the TBST binding (phage input) and washing buffer and the associated stringency were increased from 0.1% in the first round over 0.3% in the second to 0.5% in the third round. After the last biopanning round – in this case the third – no further amplification of the eluted phages was carried out.

#### 2.6.2 Biopanning on carrier material and Eu^3+^ functionalized carrier material with glycine-HCl-ultrasonic elution

A further biopanning was conducted in parallel on two different materials, on Eu^3+^-loaded NTA agarose beads and on unloaded NTA agarose beads (negative selection panning), in order to later distinguish true good binders from non-specific binders by the ratio of the same sequences that occurred with the two materials. For that, as already described, 200 μL settling volumes of the prepared Eu-NTA agarose beads as well as the NTA agarose beads were washed twice with TBST buffer (0.1% (v/v) Tween 20 in the first, 0.5% in the second round). In a following step, the targets were mixed with a Ph.D.-12 phage library dilution with a concentration of 10^11^ pfu per mL (of which 10 µL were kept for the input titration). After 1 hour of incubation in an overhead shaker (99 rpm), the beads were washed 25 times with TBST buffer and four times with 0.2 M glycine-HCl (pH 2.2). Well-bound phage clones were eluted from the target material by adding 250 µL of glycine-HCl and incubating in an ultrasonic bath (50 kHz) for 10 min, followed by neutralization of the supernatant with 44 µL of 1 M TRIS buffer (pH 9.1). The further procedure with eluates and amplicons corresponded to section 2.6.1. Two selection rounds were performed, whereby in the second round the amplified eluate from the Eu-NTA agarose beads panning was again incubated with (fresh) Eu-NTA agarose beads. In parallel the amplified eluate of the unloaded beads panning was incubated with (fresh) NTA agarose beads.

#### 2.6.3 Biopanning using Eu^3+^-loaded phage on carrier material with biological and glycine-HCl elution and subsequent negative selection step

**FIGURE 2:**
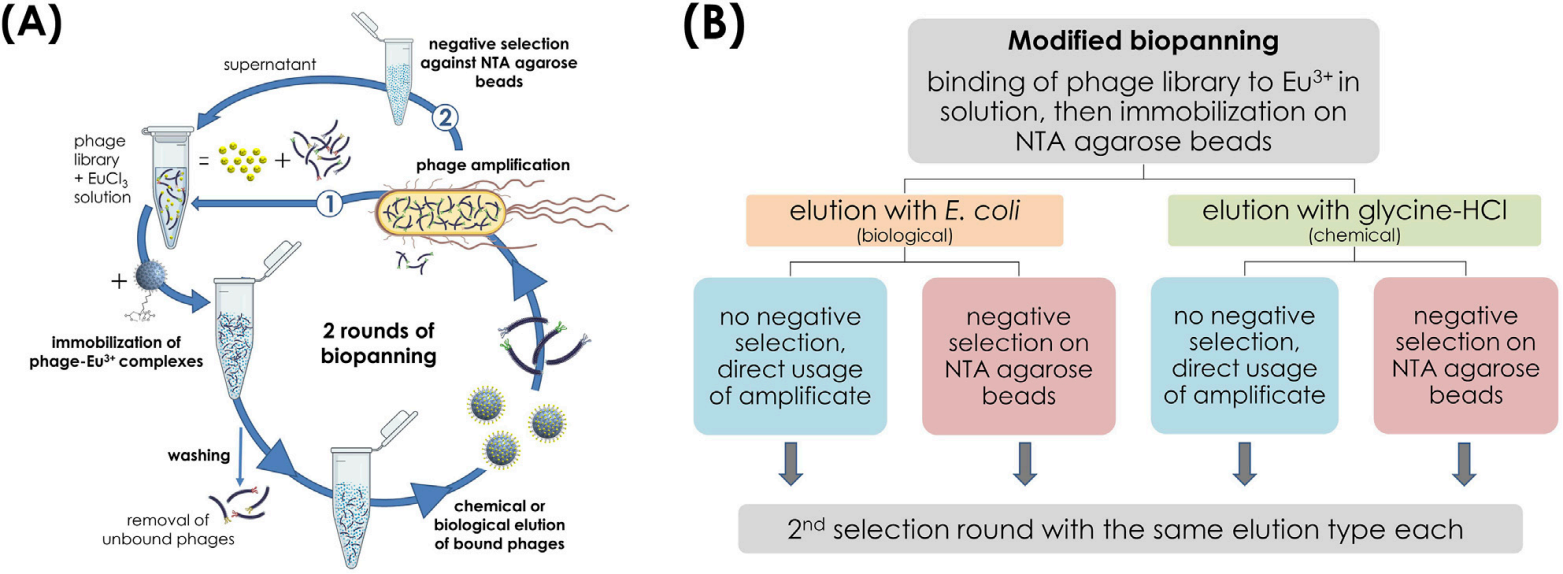
Modified biopanning with library target incubation first before immobilization of phage-target complexes on NTA agarose beads. **(A)** Schematic illustration of the biopanning process with two different types of elution, each without (1) and with (2) subsequent negative screening. **(B)** Corresponding process flow sheet.

Another biopanning was slightly modified regarding its order (Figure 2), based on Yun et al. (2019). For this, the phage library was first incubated with a 10 mM EuCl_3_ solution for 1 h at room temperature so that the Eu^3+^ ions were freely present in solution. Then, the phage-Eu^3+^ complexes were immobilized to the NTA agarose beads overnight at 4°C. Unbound phages were removed by washing 10 times with TBST buffer containing 0.1% (v/v) Tween 20. From the washed beads, three reference samples of 60 µL each were taken for determination of the Eu^3+^ content and remaining beads were divided into two tubes. One batch was eluted biologically by the addition of *E. coli* cells, the other batch chemically by glycine-HCl (0.2 M, pH 2.2). The amplified eluate from the first selection round was divided in each case, with one-half used directly for the next round and the other subjected to a negative selection (depletion) step. For this, 10^11^ pfu per mL of the amplicon were incubated with 200 µL unloaded NTA agarose beads for 1 h at room temperature and the supernatant was used for the next round. The same elution type was maintained for each eluate fraction in the two selection rounds, and TBST buffer containing 0.5% (v/v) Tween 20 was used for stringency in the second round. The further procedure with eluates and amplicons can be found in section 2.6.1.

### 2.7 Identification of peptides sequences by Sanger sequencing

To validate the success of each biopanning experiment discussed in this study individual clones from the last round of selection were analysed by Sanger sequencing. Single plaques of infected host bacteria were picked from titer plates and transferred to 50 μL TBS, allowing phages to diffuse overnight at 4°C. Residual agar and cells were removed by centrifugation (10,000 × g, 10 min, 4°C). Phage particles were stored at 4°C for further characterization. To identify the displayed peptide sequence of individual bacteriophage clones, the phage particle solution was used as the template for a polymerase chain reaction (PCR) with the oligonucleotide primer pair 5′-GCA​ACT​ATC​GGT​ATC​AAG​CT-3′ (forward) and 5′-CCC​TCA​TAG​TTA​GCG​TAA​CG-3′ (reverse). PCR was performed using Taq DNA Polymerase with ThermoPol^®^ Buffer (New England Biolabs GmbH, Frankfurt am Main, Germany) following the manufacturer’s protocol: initial denaturation (120 s, 95°C), 35 cycles of denaturation (30 s, 95°C), annealing (30 s, 55°C), elongation (45 s, 72°C), and a final elongation (120 s, 72°C). The resulting PCR product was sequenced via Sanger sequencing (Eurofins Genomics, Köln, Germany) using the primer 5′-CCC​TCA​TAG​TTA​GCG​TAA​CG-3′.

### 2.8 Identification of peptide sequence motifs by NGS

To avoid bias in the peptide sequences due to amplification advantages of some phages as well as random selection of phage clones from clone plates, the phage eluates from the last round of selection were analysed using Illumina-NGS. This sequencing method has a higher throughput and thus allows a more holistic view of a large number of strongly bound and eluted phage variants. In preparation for NGS samples, the peptide-coding inserts of the genomes of the eluted phages were first amplified by PCR using the oligonucleotide primer pair 5′ATT​ATT​CGC​AAT​TCC​TTT​AGT​G-3′ (forward) and 5′CTT​TCC​AGA​CGT​TAG​TAA​ATG-3′ (reverse) at a concentration of 0.5 µM each and Phusion Hot Start II High-Fidelity DNA Polymerase (Thermo Fisher Scientific GmbH, Dreieich, Germany) at 0.015 U/µL. The remaining components were used according to the manufacturer’s instructions, with an additional 3% Dimethylsulfoxid (DMSO) added. DNA template (phage eluate) was used at 1.25 µL per 50 µL-reaction volume. For difficult templates, the 5X Phusion HF Buffer was replaced with the 5X Phusion GC Buffer (Thermo Fisher Scientific GmbH, Dreieich, Germany). The following temperature-time program was used: initial denaturation of 180 s at 98°C; 40 cycles of 10 s and 98°C denaturation, 60 s and 60.5°C annealing, and 30 s and 72°C elongation; and a final elongation of 300 s at 72°C. The PCR products were separated by agarose gel electrophoresis (2%), the dominant fragments of the correct size (161 bp) were cut out (see Supplementary Figure S1) and purified using the Wizard SV Gel and PCR Clean-Up System kit (Promega GmbH, Walldorf, Germany). DNA concentration was determined using the Qubit 4 Fluorometer with the Qubit 1x dsDNA HS Assay Kit (Thermo Fisher Scientific GmbH). NGSelect paired-end Illumina sequencing with 2 × 150 bp was performed externally by Eurofins Genomics Germany GmbH, Ebersberg, Germany. The 5 million read pairs per sample in format of FASTQ files were analysed using an in-house script. Known biases in the library (*e.g.*, overrepresentation of wild-type clones, stop codons) were addressed by filtering out sequences that did not match the expected peptide library format. This ensured that only valid peptides were considered for frequency calculation. For this, the size of the fraction, which is either the eluate, original or amplified phage library, was first determined as the total number of reads without read count 1, using this value to calculate proportions. For each peptide sequence, its relative frequency within in the original library was determined using the formula:

$$\text{Relative frequency in original library }\left(\text{Rel}.\text{ freq}.\text{ in ori}.\text{ lib}.\right)=\frac{\text{number}\;\text{of}\;\text{reads}\;\text{for}\;a\;\text{specific}\;\text{sequence}}{\text{total}\;\text{number}\;\text{of}\;\text{reads}\;\text{in}\;\text{original}\;\text{library}}$$

For sequences detected in the original library, their relative frequency is directly reported as the calculated ratio. For sequences not detected in the original library, the relative frequency is marked as “n.a.” (not available), since it cannot be determined from the data. For the calculation of enrichment factors, the relative frequencies of both the main biopanning elution fraction and the corresponding negative selection fraction were determined, using the formula:

$$\text{Relative frequency in respective fraction}=\frac{\text{number}\;\text{of}\;\text{reads}\;\text{for}\;a\;\text{specific}\;\text{sequence}}{\text{total}\;\text{number}\;\text{of}\;\text{reads}\;\text{in}\;\text{respective}\;\text{fraction}}$$

Relating these two values to each other gives the enrichment factor for one specific sequence, as shown in the following formula:

$$\text{enrichment factor}=\frac{\text{relative}\;\text{frequency}\;\text{in}\;\text{main}\;\text{biopanning}\;\text{elution}\;\text{fraction}}{\text{relative}\;\text{frequency}\;\text{in}\;\text{negative}\;\text{selection}\;\text{fraction}}$$

The amplification advantage factors were calculated in the same way as the enrichment factors, using the ratio of the relative frequencies of amplified to original library instead of eluate to negative selection fraction:

$$\text{amplification advantage factor}=\frac{\text{relative}\;\text{frequency}\;\text{in}\;\text{amplified}\;\text{library}\;}{\text{relative}\;\text{frequency}\;\text{in}\;\text{original}\;\text{library}}$$

Relative frequencies are expressed as small fractions, e.g., 2.5 × 10^−6^, due to the high diversity of the library or elution fraction, respectively. For this example, it means that for every 1,000,000 reads, approximately 2.5 reads correspond to that specific peptide sequence.

### 2.9 Eu^3+^ binding studies using time-resolved laser fluorescence spectroscopy

In order to analyse the affinity of the selected peptides towards Eu^3+^, each peptide was titrated into a solution of EuCl_3_ and excited using a laser, the emitted light of which was recorded in 21 time-shifted spectra. For each peptide, three titration series (=triplicate) consisting of 20 titration steps with increasing peptide concentration were measured.

Peptides (Tables 1, 2) as well as CaM-EF4 (DIDGDQVNYEE) that served as reference peptide were synthesized via solid-phase peptide synthesis (SPPS) and obtained from DGpeptides (Co., Ltd.; Hangzhou, CN) as lyophilized TFA salts with a purity of ≥85%. To prepare peptide stock solutions, 4–6 mg of lyophilized peptide powder was dissolved in an appropriate volume of ultrapure water (Merck KGaA, Darmstadt, Germany) to achieve a stock concentration of 5 mM. The peptide concentration was measured using a NanoDrop spectrophotometer (Life Technologies GmbH, Darmstadt, Germany). Potassium chloride was added to a final concentration of 100 mM, and the pH was adjusted to 5.2 using NaOH. The stock solution was then diluted to a final peptide concentration of 2.5 mM.

**TABLE 1:** Peptide sequences selected for synthesis and affinity analysis from several independent biopanning experiments and their enrichment factors, possible amplification advantages, relative frequency in original library, theoretical pI (calculated with Expasy ProtParam) and polystyrene surface binding probability [PSBP, calculated with SAROTUP (HLAB Co, 2025)].

Peptide	Sequence^a^	pI	Rel. freq. in ori. lib	Amp. advant	Enrichment factors					PSBP
					(A)	(B)	(C)	(D1)	(D2)	
GC1	VHHVAGNALQPF	6.89	n.a	n.a	2	2	762	145	118	0.46
GC2	SSQMVTNHQFVL	6.46	2.5*10^–6^	n.a	0	3	176	241	213	0.29
GC3	SIHSVTKGRYPV	9.99	n.a	n.a	0	n.a.	n.a.	4	31	0.32
GC4	HDPRMEHSLPKS	6.92	5.8*10^–6^	0.3	4	4	1	49	1,037	0.04
GC5	TLAHHPVSISHS	6.73	n.a	n.a	1	6	390	179	104	0.33
GC7	VAHVDGTPRLRN	9.58	n.a	n.a	49	47	29	155	127	0.08
GC8	ARSLEPAPSRHS^b^	9.65	2.8*10^–4^	1.6	3	2	8	75	90	0.61
GC9	EALTVNIKREME^b^	4.79	3.1*10^–4^	1.5	2	4	5	123	142	0.15
RB-Ar-8	SIHSVTKGWYPV	8.33	Similar to GC3, differ only in one amino acid: W instead of R (Braun et al., 2020)							0.61

N.a. is not available since this sequence was not sequenced by NGS within one of the required fractions used for calculation of the corresponding factor. (A) Main biopanning against the target with biological elution = ratio MB/original library.

(B) Main biopanning against the target with glycine-HCl, elution = ratio MB/original library.

(C) Parallel biopanning against the target (main biopanning = MB) as well as unloaded NTA agarose beads (parallel negative selection panning = PNSP) with ultrasound elution = ratio MB/PNSP.

(D) modified biopanning with the amplified eluate divided into two fractions, one incubated against NTA agarose beads in a negative selection step (NSS) and one used directly for the next round of selection (see methods) with either biological (D1) or chemical (D2) elution = ratio w/wo NSS.

^a^ Green, positively charged (basic) amino acids; red, negatively charged (acidic) amino acids; orange, uncharged polar amino acids; blue, nonpolar aliphatic amino acids; purple, proline as secondary structure breaker; black, aromatic amino acids.

^b^ identified in biopanning with *E. coli* elution (see section 2.6.1) via Sanger sequencing; see Supplementary Tables S3, S4; (Claus, 2020).

**TABLE 2:** Original enriched peptide sequences GC1 and GC3 were modified by substitution of certain amino acids and/or incorporating additional (acidic) amino acids. The peptides were synthesized with GGGS linker, which connects peptide with the minor coat protein on the bacteriophage.

Name of peptide variant	Original/modified	Sequence
GC1	original	VHHVAGNALQPFGGGS
GC1-A/D	modified	VHHVDGNDLQPFGGGS
GC1+D+E	modified	DVHHVDGNDLQPFEGGGS
GC3	original	SIHSVTKGRYPVGGGS
GC3-V5/D	modified	SIHSDTKGRYPVGGGS
GC3+D+E	modified	DSIHSDVTKDGRYPVEGGGS

For each titration series, 500 µL of a 10 μM EuCl_3_ solution (in 100 mM KCl, pH 5.2) was placed in a quartz glass cuvette. A 2.5 mM peptide stock solution (in 100 mM KCl, pH 5.2) including 10 μM EuCl_3_ (to avoid dilution effects) was titrated to the EuCl_3_ solution with a dynamic increase in concentration (measurement 1 was without any peptide, see Supplementary Table S1).

For TRLFS, a Nd:YAG laser-pumped optical parametric oscillator (OPO, NT230, Ekspla) with ∼5 ns pulse and a wavelength of 394 nm was used to excite the sample in a quartz glass cuvette. An optical fibre was used to transmit the emitted light to the spectrometer (SR-303i-A, Andor Technology). A grating with 300 lines per mm generated monochromatic light, which was detected with a cooled (−30°C) ICCD camera (Andor iStar, DH320T-18U-63). The initial delay was set to 5 μs, gate width to 200 µs, slit width to 200 µm and gain to 4,095. Eight accumulations were gathered. Baseline correction was performed by Andor Solis software (Andor Technology). The step size for 21 spectra was calculated from the formula 3e^+6^+(3e^+6x^) μs. A Peltier element (TC1 temperature controller, Quantum Northwest) controlled the sample temperature of 25°C and the mixing of the sample (1,200 rpm) using a magnetic stir bar. The three-dimensional fluorescence data obtained were analysed using PARAllel FACtor analysis (PARAFAC).

## 3 Results

### 3.1 Biopanning experiments

The goal of the biopanning experiments described was to identify peptide sequence motifs with an affinity for Eu^3+^ ions. This was achieved by screening the commercially available Ph.D.-12 Phage Display Peptide Library (NEB) displaying randomized dodecamer peptides on the pIII phage minor coat protein. In order to retain the Eu^3+^ binding phages, it was necessary to immobilize our target Eu^3+^ on a solid matrix such as NTA agarose beads. The Eu^3+^ content of the NTA agarose was determined by ICP-MS after elution with 1 M HNO_3_ in three reference samples. This resulted in a loading of 120 μg Eu^3+^/100 μL bead volume. Thus, 240 μg Eu^3+^ were available as target material for each biopanning round, in which approximately 200 μL beads were provided.

Several independent biopannings were carried out using different elution methods. In the first panning strategy (Figure 1), the phage library was only incubated with the Eu-NTA agarose beads. In contrast, in the second panning strategy, the phage library was incubated with the Eu-NTA agarose beads as target material (main biopanning) and with the NTA agarose beads as negative control (negative selection panning) in parallel. The negative selection panning involves the binding of phages to plastic and/or the immobilisation material (NTA agarose beads), so that the phage library is cleared of non-specific binders (Nian et al., 2010; New England Biolabs, 2024). However, a negative selection step before target binding was deliberately avoided in order to prevent the removal of potentially good binders and thus premature depletion of the library. Instead, a parallel approach was used to screen for target-unspecific peptide variants (TUP), which were then compared with the peptide sequences from the main biopanning and eliminated. In a third panning strategy (Figure 2), the order of the classic biopanning process was changed slightly. In a first step, the bacteriophage library was incubated with the EuCl_3_ solution before the Eu^3+^-phage complexes were immobilized on the matrix. The amplified eluate was divided into two fractions, one of which was subjected to a negative selection step against unloaded NTA agarose beads before being used for the next round. The other fraction was used directly as input for the next round of selection.

### 3.2 PSD enrichment and NGS identification of Eu^3+^ binding peptide sequence motifs

NGS was applied to identify the peptide inserts of the bacteriophages contained in the eluate of the final selection round. An in-house bioinformatics script was used to process the raw sequencing data (FASTQ files) so that enriched peptide sequences and their absolute abundances could be extracted. These absolute frequencies were set in relation to the reads per fraction in order to derive the relative abundances, which finally allow a comparison of the sequences with each other. The enrichment factor, which assesses whether a peptide variant is target-specific or interacts non-specifically with the NTA agarose beads, was determined by comparing the relative abundances in the elution fraction to those in the corresponding negative control. This negative control could either involve a parallel negative selection panning (ratio main biopanning/negative selection panning) or no subsequent negative selection step after elution (ratio with/without subsequent negative selection step), depending on the experimental setup (see visualization in Supplementary Figures S5, S6). To evaluate whether a sequence motif accumulated due to its affinity for the target or was influenced by amplification advantages during the selection rounds, the ratio of the relative abundances in the amplified library to those in the original library was calculated. Ratios greater than one indicate an amplification advantage, which should be considered when interpreting the enrichment of specific peptide sequences.

Since the parallel biopanning against both Eu^3+^-loaded and unloaded NTA agarose beads as well as the modified biopanning with and without a negative selection step on the amplified eluate were each performed with the same phage library (same lot number) but completely independently and with different elution types, the identified peptide sequences were screened for repeated occurrence in several different eluate fractions.

For the synthesis and affinity analysis of the peptides without phage construct, a small selection of interesting peptides was chosen from the huge amount of enriched and NGS-sequenced peptides (Table 1). The selection criteria included: (Schüler et al., 2011) strong enrichment in multiple independent biopannings or eluate fractions, as determined by sequencing data (Adler and Müller, 2014); consistent amplification across rounds, indicating robust phage growth (≤1); and (Fricker, 2006) minimal non-specific binding to plastic surfaces (≤0.5), calculated with SAROTUP (HLAB Co, 2025). The peptides identified by Sanger sequencing and selected for further analysis did not undergo the same selection criteria. While the high data density of NGS data allows selection based on specific criteria, the limited clone size in Sanger sequencing results is insufficient for such an approach. Some of the NGS-sequenced peptide variants are quite similar in their amino acid sequence, such as the two possible progeny sequences of VHHVAGNALQPF: VHHVAGNVLQPF and VHHVAGNALQPS (the latter two are not shown in Table 1 due to space constraints; only synthesized and tested sequences are included). The original sequence VHHVAGNALQPF shows an increased enrichment inthree independent eluates [Table 1, columns (C), (D1) and (D2)] compared to the respective negative control, either directly in parallel or as in the modified panning afterwards. In ultrasound elution it is even the most enriched peptide variant. Many of the other identified peptide sequences are rich in basic and neutral polar amino acids like serine, asparagine or glutamine and often contain at least one proline and/or acidic amino acid. Also notable are short motifs found in more than one sequence, such as Sx_1-2_HS (where x is any amino acid) and VxHV. Moreover, the sequence SIHSVTKGRYPV (GC3) was already found in a previous biopanning against arsenic oxyanions (Braun et al., 2020). Sequence SIHSVTKGWYPV (RB-Ar-8), also enriched against arsenic oxyanions (Braun et al., 2020), differs from GC3 in only one amino acid at position 9 (tryptophan instead of arginine), which is why this peptide was also investigated in more detail. Identified and promising peptides shown in Table 1 were synthesized as linear peptides in a solid phase synthesis and dissolved in distilled water. Peptide GC2 was not soluble in water but only in 20% acetic acid and could therefore not be tested, as acetic acid itself interacts with Eu^3+^.

### 3.3 Binding study of PSD-enriched and NGS-identified peptides using TRLFS

Since Eu^3+^ is a fluorescent element, the binding affinity of the selected peptides towards Eu^3+^ was assessed by TRLFS. For this, changes in the Eu spectrum, in particular in the ^7^F_1_/^7^F_2_ peak intensity ratio, were determined. A titration series with a peptide excess of up to 100-fold relative to Eu^3+^ was used to detect even weak affinities. The sequences of GC1 and GC3 were subsequently modified at specific positions to improve binding affinity. These modifications were based on two criteria: the peptides’ high enrichment levels during biopanning and insights from initial binding tests (data not shown), which indicated potential interactions. By mimicking natural occurring Ln-binding molecules, the modifications targeted specific acidic amino acid residues believed to enhance Eu^3+^ binding by facilitating ionic interactions and taking into account structural considerations as discussed before. The goal was to form binding pockets and/or to replace alanine or valine, which were unexpected to interact with Eu^3+^. Derivates of the peptides were synthesized to assess the role of specific amino acids in Eu^3+^ binding (Table 2). The original peptides GC1 and GC3 as well as their modified versions were synthesized with a GGGS linker in order to have the possibility to easier immobilize them on a matrix for adsorption experiments in case of good affinity. We tested GC1 and GC3 with and without the GGGS linker but found no significant improvement or reduction in binding affinity (data not shown).

**FIGURE 3:**
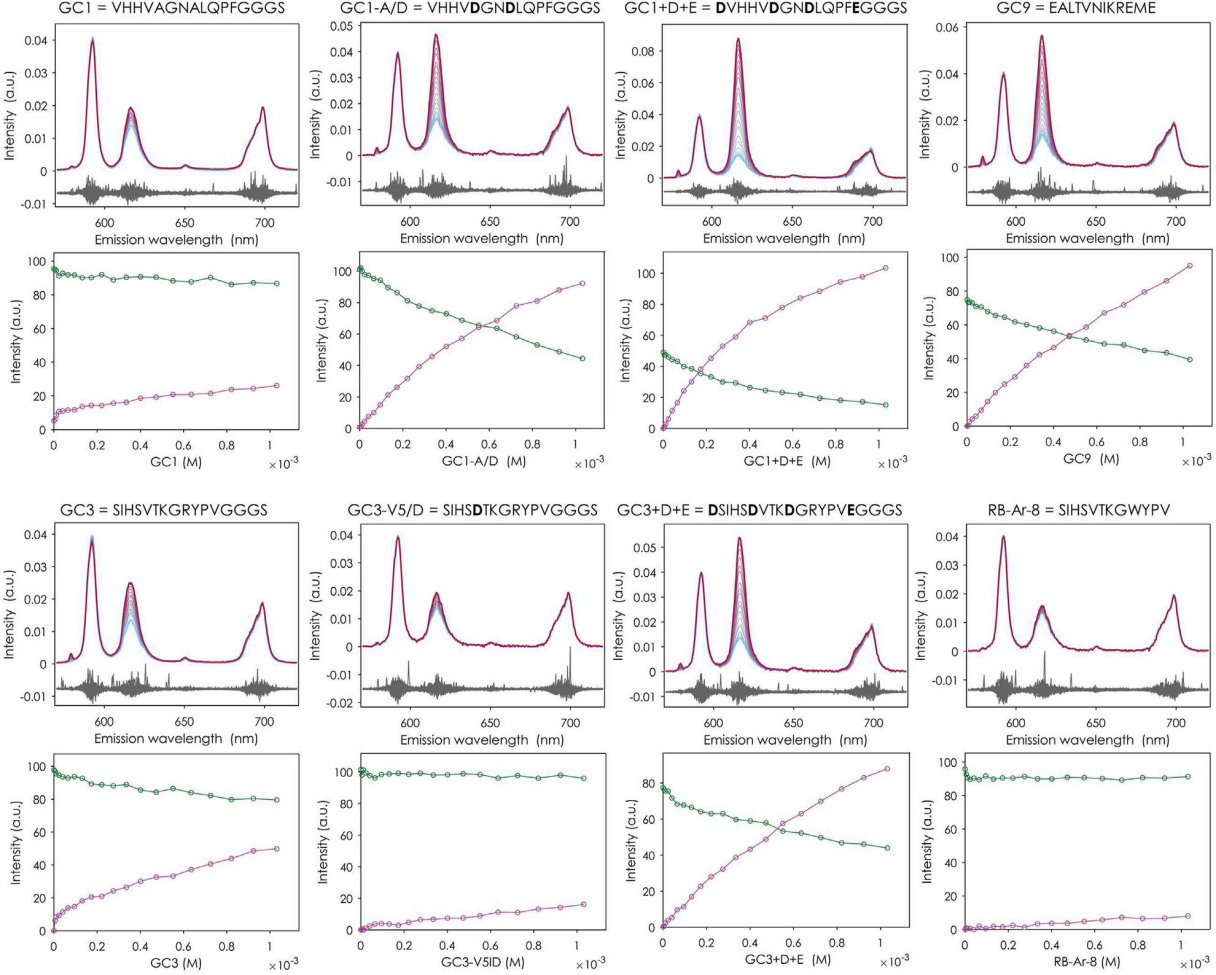
Eu^3+^ complexation studies of some selected peptides with TRLFS. t_0_ fluorescence emission spectra (top each) and distribution diagrams derived from them (bottom each) with Eu^3+^ aquo ion as green line and 1:1 complex as purple line. Titration of 0–1,031 µM peptide to 10 µM EuCl_3_ in 20 steps, pH 5.2, background 100 mM KCl. Not all peptides enriched in biopannings (from Table 1) are shown here. See Supplementary Figures S2–S4.

For eight of the twelve peptides tested (GC1, GC1-A/D, GC1+D + E, GC3, GC3+D + E, GC4, GC8, GC9), changes were observed in the emission spectrum of the Eu^3+^ aquo ion. Specifically, the intensity of the ^7^F_2_ peak increased relative to the ^7^F_1_ peak, which is characteristic of Eu^3+^ binding to the peptides and indicative of complexation (Figure 3).

Peptide GC1 and its modified variants demonstrate how the substitution of specific residues with acidic amino acids can significantly enhance binding affinity. The originally selected GC1 showed very low binding, characterized by the only slightly increased intensity of the ^7^F_2_ peak compared to that in the spectrum of the Eu^3+^ aquo ion. The modified variant GC1-A/D, in which the alanines were replaced by aspartic acids, already showed improved Eu^3+^ binding. In the variant with additional N- and C-terminal aspartic and glutamic acids even a reversal of the peak ratio could be observed. When examining the distribution of different species in the system with increasing peptide concentration, only a small decrease in the intensity of the free Eu^3+^ ion and a slight increase in the 1:1 complex can be observed for GC1. For the two modified variants of GC1, in contrast, a strong decrease or increase of the corresponding species can be observed, indicating an enhanced complexation of Eu^3+^.

This can only be applied to a limited extent to the GC3 peptide and its modified versions. Although GC3 exhibited a higher affinity for Eu^3+^ than GC1, replacing the valine at position five with aspartic acid (GC3-V5/D) did not improve binding. On the contrary, it resulted in an affinity reduction. The addition of acidic amino acids in GC3+D+E led to a higher Eu^3+^ complexation, but not as high as in GC1+D+E.

Among the unmodified peptides enriched in the biopanning experiments, GC9 was the one with the highest affinity for Eu^3+^. Its t_0_ spectra and species distribution diagram are similar to those of GC3+D+E, although it has one less acidic residue than the modified variant of GC3.

Peptide RB-Ar-8, already selected in a previous biopanning against arsenic oxyanions (Braun et al., 2020) and differing from GC3 only in one amino acid (tryptophan instead of arginine), showed no change in the spectrum and thus no binding to Eu^3+^, suggesting that the arginine at position nine in peptide GC3 plays an essential role in the binding. No binding affinity was found for GC5 and GC7 either.

## 4 Discussion

CaM-EF4 (DIDGDQVNYEE), which serves as our reference peptide, is one of the four EF-hands of CaM, a protein known from nature that not only binds its actual target metal calcium, but also has shown to have an affinity for Ln (Drobot et al., 2019; Mulqueen et al., 1985; Kilhoffer et al., 1980). CaM-EF4 consists of 12 amino acids, five of which have acidic properties. Some of these acidic residues are responsible for the strong Ln^3+^ affinity with a K_D_ of 6.8 × 10^−6^ M for Eu^3+^ (Perevedentseva et al., 2024). At the same time, however, the acidic amino acids and their coordination to the metal ion through electrostatic interactions and carboxylate groups could also be responsible for the lack of selectivity within the Ln series. This is because such interactions are not highly specific and can occur similarly with other Ln, leading to reduced discrimination. In addition to CaM, there are other very similar proteins such as LanM or LanP, which can bind Ln with high affinity. However, the selectivity of these proteins for specific Ln has not yet been fully studied. For LanM only a slight increase in affinity with a decrease in the Ln^3+^ coordination sphere was demonstrated (Prejanò et al., 2023), indicating missing selectivity for one specific Ln. In this first study, we investigated the hypothesis that PSD as kind of directed evolution can be used to identify previously unknown peptides that can bind Eu^3+^ with a comparably high affinity but with higher selectivity. Using PSD, peptides can be selected under different pH conditions or in the presence of competitive binders, such as other heavy metals that may be present in wastewater. For comparability with CaM-EF4, the Ph.D.™-12 phage display peptide library with an equal length of 12 amino acids was chosen as input for the screening. The randomized approach allowed us to identify high-affinity ligands from a diverse peptide library, without pre-selecting for specific features such as amino acid composition or the presence of acidic amino acids. With this, we have succeeded in identifying novel peptides, some of which have increased affinities for Eu^3+^.

### 4.1 Eu^3+^ as target for biopanning

For the biopanning selection process, bacteriophages that bind strongly to the target must be separated from weak or non-binding ones. While insoluble targets like inorganic particles can be directly used, soluble targets such as ions require reliable immobilization on a matrix. This ensures that strongly binding phages or phage-target complexes remain on the solid phase, while weak and non-specific binders are removed through repeated washing. Finally, the most strongly bound phages are eluted from the matrix. The challenge here is not to immobilize a sufficiently high number of ions. With 240 μg Eu^3+^ or approximately 9.5 × 10^17^ Eu^3+^ available per selection round to bind 1 × 10^11^ phage particles, there was a clear excess of Eu^3+^, so that there should have been sufficient target ions for each phage clone. However, during metal ion immobilization, some of the metal binding sites are already occupied. In the case of Eu^3+^ and NTA theoretically four of the nine binding sites are occupied and thus not available for potential interaction with peptides. In addition, target immobilization in combination with affinity selection such as biopanning carries the risk that potential superbinders strip the Eu^3+^ from the NTA and are thus lost in the washing step. All these reasons justify a well-planned biopanning for Eu^3+^ as well as the combination of various protocols with respect to the target presentation, interaction environment as well as the type of binding.

### 4.2 Enrichment of Eu^3+^ binding phage clones

For the enrichment and identification of Eu^3+^ binding phage clones in this preliminary study, a phage peptide library was screened that displays 12 amino acid long randomized peptides on the five pIII minor coat proteins of the M13 bacteriophage with a diversity of about 10^9^ single sequences. Considering our immobilized target, a pIII phage library seemed to be a better choice than a pVIII phage library. While pVIII phages display up to 270 peptide copies along the entire 1 µm length of the filamentous M13 phage (Kay and Hoess, 1996), this high density can lead to target clogging, making it difficult for peptides to effectively bind the immobilized Eu^3+^ ions. In contrast, the pIII phages display only five peptide copies at the proximal end, allowing more precise interactions with the target and reducing competition among phage variants. Although pVIII has more peptide copies and thus theoretically a multiple binding strength, the successful selection of phages with only five copies in the pIII system suggests strong and genuine target binding, making a pIII phage library more suitable for this study’s goal of identifying high-affinity Eu^3+^ binding peptides.

Furthermore, we refrained from blocking the target material with bovine serum albumin (BSA) prior to panning, which is often done in case of other targets (e.g., organic targets, insoluble targets) to avoid unspecific interactions with the target, as we wanted to prevent complete masking of the target and thus a lack of interaction with the phages. The selection pressure on the phages was increased with each round by rising the concentration of Tween 20 in the TBST buffer. As a surfactant, Tween 20 reduces the interfacial tension between two phases, which is why it becomes more difficult for the phages to attach to the target and to the resin and plastic surface as its concentration increases. As a result, only the strong-binding phages are still able to bind to the target material. In addition, negative selection prior to the binding step was avoided due to the risk of premature depletion of the library and thus premature exclusion of potentially strong-binding peptide variants. Instead, such a negative selection against the unloaded NTA agarose beads to remove TUPs was performed either in parallel or after the main biopanning following the propagation of the eluted phages.

### 4.3 Peptide sequence identification and evaluation using NGS

For sequence identification of the peptide inserts of a large number (five million read pairs) of bacteriophages contained in the eluate fractions of the last selection round, we decided to use NGS in addition to Sanger sequencing (see Supplementary Tables S3, S4). NGS gives us a more holistic view of all strongly bound and eluted phage variants compared to Sanger sequencing of randomly isolated single phage clones. By comparing the relative sequence abundances of main biopanning and negative selection panning fractions, as well as of the naïve and amplified phage library, enrichment factors or amplification advantages of individual sequences can be calculated, and TUPs can be eliminated. The ratio of the amplified to naïve bacteriophage library should always be considered as an amplification advantage factor when evaluating frequent peptide sequences. Since certain phage clones have a propagation advantage and others a disadvantage and are therefore more or less strongly represented in the input phage solution for the next selection round, the amplification steps between rounds sometimes favour peptides that are not necessarily the best target binders (Derda et al., 2011). Phage clones with peptide sequences that have a growth advantage [propagation-advantaged phages (PAPs)] will always displace phage clones with peptide sequences that amplify more slowly, although both may have equally good binding properties to the target material (Rodi et al., 2002). The reasons for such an advantage or disadvantage are manifold and can occur in any amplification step. On the one hand, some peptide structures are obstructive for the infection of the host with the phage or for the packaging of the fusion protein (Rodi et al., 1999), so that this phage variant is less strongly amplified and other phage clones therefore occur more frequently in the amplificate. However, other metabolic and structural effects are also a limiting factor, such as the fact that each of the 20 amino acids is encoded by a different number of codons (Cwirla et al., 1990) or that advantageous mutations may be present in the regulatory region of the phage genes (Brammer et al., 2008). Derda et al. (2011) reported that about 70% of phage clones can be eliminated in a single amplification step. A biopanning with many rounds of selection is therefore not necessarily the best solution for selecting strong binders, as the sequence diversity is reduced enormously. The calculated enrichment factor, as well as the amplification advantage factor and the associated reduction of TUPs and PAPs, considerably increase the theoretical probability of finding peptide variants with a real affinity to the target.

From the large data set of NGS-analysed enriched sequences, some interesting ones were selected and characterized with regard to their amino acid composition. For this, besides a strong enrichment factor in several different elution fractions, we took also into account amplification advantages (PAPs) as well as the probability of binding plastic and thus being a non-specific binder (TUPs). As mentioned previously, we identified the highly enriched sequence VHHVAGNALQPF, along with two very similar sequences: VHHVAGNVLQPF and VHHVAGNALQPS. It remains unclear whether these differences represent true sequence variants contributing to a conserved motif important for target binding or are simply the result of sequencing or PCR errors during the experiment. If these sequences are genuine variants, they may form a cluster of related motifs with enhanced binding potential. Alternatively, if the variations are due to errors, the reads from these sequences should be attributed to the original sequence, increasing its overall enrichment factor. Further analysis would be required to distinguish between these possibilities. Many of the peptide sequences that are most enriched in multiple independent biopannings have numerous basic as well as neutral polar amino acids like serine, asparagine and glutamine and at least one proline and/or one acidic residue. The sequences richest in acidic amino acids are HDPRMEHSLPKS and EALTVNIKREME. Short motifs that occurred in more than one sequence were identified as Sx_1-2_HS (SIHSVTKGRYPV, TLAHHPVSISHS, ARSLEPAPSRHS) and VxHV (VHHVAGNALQPF, VAHVDGTPRLRN), where x represents any amino acid. Since the motif-bearing sequence SIHSVTKGRYPV (GC3), as well as SIHSVTKGWYPV (RB-Ar-8), which differs from GC3 in only one amino acid (W instead of R), could already be enriched against arsenic oxyanions in a previous biopanning (Braun et al., 2020), it was assumed that the motif SxHS contribute substantially to metal binding. However, this hypothesis could not be proven in this study. Our experiments showed a moderate binding of GC3, but no binding for RB-Ar-8, indicating that the SxHS motif that both sequences have in common is not responsible for Eu^3+^ binding. This suggests other factors, such as sequence context or peptide conformation, play a larger role. While acidic amino acids with their carboxyl groups play an important role in the interaction of Ln^3+^ ions (Hosokawa et al., 2022; Legendziewicz et al., 1980), basic amino acids, in particular histidine, but also lysine, are better known for their affinity to transition metals like nickel (Hochuli et al., 1988) or copper (Curtis et al., 2017), and interact with the amino groups of their side chain. The presence of basic amino acids was, therefore, unexpected, but can be justified by the presence of the NTA group in the resin. The bond between the carboxyl groups of NTA and Eu^3+^ ions is nitrogen-stabilized by the side chain amino group of the basic residues, which could explain why peptides containing basic amino acids have been enriched during the panning.

Generally, the experimental setup could serve the purpose to identify peptides for any metal ion target. In other studies (transition) metal ions such as Ni^2+^ (Pagano et al., 2015a; Kay and Hoess, 1996), Zn^2+^ (Derda et al., 2011), or Ga^3+^ (Gonzalez et al., 2014) were immobilized on solid matrices, and biopanning experiments demonstrated good sequence enrichment and strong metal affinities for the selected peptides. However, the ion radius and available binding sites of a metal ion effect the immobilization mechanism. The interaction of transition metal ions with NTA or IDA differs from that of Eu^3+^ ions. The high affinity of the carboxyl groups in NTA for Ln^3+^ ions means that Eu^3+^ may bind more tightly to NTA compared to Ni^2+^ or Zn^2+^, assuming that more binding sites are occupied. Following this theory, the enriched phages or peptides are then Eu^3+^-NTA-adapted variants, which could potentially act as moderate to weak binders for free Eu^3+^, as they utilize only a portion of the available binding sites for the interaction. This highlights the importance of considering how the immobilization method influences target presentation, as it can significantly affect the outcome of biopanning experiments, particularly when using NTA- or IDA-based matrices.

Nevertheless, we succeeded in enriching interesting sequences and since, as already mentioned, we wanted to use a randomized approach with regard to the amino acid composition, the selected sequences (Table 1) were synthesized and investigated for their affinity.

### 4.4 TRLFS binding study of the most enriched dodecamer peptides

TRLFS was used to evaluate Eu^3+^ binding affinities by analysing changes in the emission spectrum, particularly the ^7^F_1_/^7^F_2_ peak intensity ratio. A 100-fold peptide excess ensured detection of weak complexes and enabled determination of europium species and affinity constants. Affinity to Eu^3+^ was detected in 8 of 12 tested peptides, highlighting the strength and efficiency of our PSD approach combined with NGS. From the original peptides selected by biopanning, GC9 (EALTVNIKREME) was found to have the highest affinity for Eu^3+^ with a K_D_ of 1.05 × 10^−3^ M (see Supplementary Table S2). Although with three acidic amino acids it has one less than GC3+D+E, the t_0_ spectra and species distribution diagrams look similar for both peptides (Figure 3). However, as previously observed for the CaM-EF4 (DIDGDQVNYEE), acidic amino acids are not always involved in target binding (Mulqueen et al., 1985). This seems to be dependent also on the three-dimensional arrangement and distance between the acidic residues. Both the reversed sequence of GC9 (EMERKINVTLAE) and CaM-EF4 have acidic amino acids at positions 1, 3, and 12. Additionally, both peptides have a valine at position 8, and a possible oxygen donor at position 7 (asparagine in GC9 and glutamine in CaM-EF4). Polar amino acids, which could also act as oxygen donors, are present at position 9 (threonine in GC9 and asparagine in CaM-EF4).

Both peptides contain nearly the same amount of hydrophobic amino acids (GC9 = five, CaM-EF4 = four), which are distributed across the sequence. While isoleucine, glycine, valine and tyrosine occur at a distance of one amino acid in CaM-EF4, the hydrophobic residues alanine, leucine, valine, isoleucine and methionine are not as evenly distributed in GC9. Due to the short length of only 12 amino acids, the peptides should not be able to fold in a manner that the binding site consists of a hydrophilic smaller shell embedded in a hydrophobic larger shell, as is the case with metal-protein binding patterns (Yamashita et al., 1990). However, a folding in which the hydrophobic residues point outwards to mimic such an effect cannot be ruled out and should be further analysed by circular dichroism.

For peptide GC1 (VHHVAGNALQPF), it could be clearly shown that the replacement of amino acids by acidic amino acids (GC1-A/D) or the incorporation of additional carboxyl group-providing amino acids (GC1+D + E, DVHHVDGNDLQPFEGGGS) increases the binding strength, reducing the K_D_ by two to three orders of magnitude (4.03 × 10^−1^ M > 1.11 × 10^−3^ M > 4.89 × 10^−4^ M, respectively). In contrast, this only applied in part to the peptide GC3 (SIHSVTKGRYPV) and its modified variants. Although GC3 already had a higher affinity (K_D_ = 1.47 × 10^−2^) than GC1 and the replacement of the residue at position five with a negatively charged amino acid was expected to result in a higher affinity, the opposite was observed. The binding could not be improved by replacing valine with aspartic acid (GC3-V5/D), suggesting that valine, as a hydrophobic amino acid at this position, probably plays an important role in the folding of GC3. The two serines in GC3 could also provide oxygen atoms for binding and could be one reason for the higher affinity of GC3 compared to GC1. For GC3+D+E (DSIHSDVTKDGRYPVEGGGS) with additionally inserted acidic amino acids, a high affinity with a K_D_ of 1.54 × 10^−3^ M could again be measured, but here the valine was kept and only an aspartic acid was inserted next to it, so that it retained its function at this position. It is also interesting that the peptide RB-Ar-8 (SIHSVTKGWYPV), which like GC3 was already identified as a possible binder in an earlier biopanning against the arsenic oxyanions and differs from GC3 only at position nine by a tryptophan instead of arginine, showed no Eu^3+^ complexation. Therefore, we infer that the basic amino acid arginine in GC3 plays a key role in binding. It can only be speculated whether tryptophan as a hydrophobic amino acid in the case of RB-Ar-8 prevents the peptide from wrapping around the Eu^3+^ ion or whether the arginine in the case of GC3 stabilises the formed complex together with the other basic amino acids histidine at position three and lysine at position seven.

In summary, eight of the 12 selected peptides showed a complexation of Eu^3+^, although in some cases with low affinity. Although none of the peptides found in the PSD approached the Eu^3+^ affinity of our reference peptide CaM-EF4, we have succeeded in identifying novel, previously unknown Eu^3+^-binding peptide sequences. These sequences and methods cannot yet exploit their full potential, as the synthetic peptides probably differ from the pIII-fused peptides in their folding. The peptides displayed by the bacteriophage are likely stabilized by the phage surface, which could improve their interaction with the environment. Thus, it may be possible that the peptides bound to the phage have a strong binding to Eu^3+^, while the synthetic, free peptides have little or no affinity–not because of the sequence itself, but because of the lack of stabilization. To overcome this, circularization of the peptide sequences could be considered. The addition of an N- and C-terminal cysteine forming a disulfide bridge can stabilize the peptide as well (Barkan et al., 2016) and thus improve the interaction with the target material (McLafferty et al., 1993). Other reasons for the reduced affinity of the peptides found compared to CaM-EF4 could lie in the way Eu^3+^ ions are immobilized, as already discussed in 4.2, and in the naïve Ph.D.-12 phage display peptide library itself. As Sloth et al. (2022) have shown, substantial deviations from randomness occur at the nucleotide, amino acid, and peptide levels in the Ph.D.-12 library. Additionally, the presence of stop codons and a high abundance of wild-type clones reduce the effective size of the naïve library. This suggests that the library may lack optimal Eu^3+^ binding motifs altogether, highlighting the need for selecting or designing a library better suited to the target.

Furthermore, an important factor to consider is the multivalency of phage-displayed peptides. In phage display, each phage presents five copies of the same peptide in a highly specific orientation, which can significantly enhance binding through cooperative interactions. In contrast, synthesized peptides are monovalent, which may lead to differences in binding affinity and behavior. This multivalency effect could explain discrepancies between phage-displayed and synthesized peptides in binding assays.

An application of the peptides for complex mining waters has not yet been tested in this study. It can be assumed that the affinity of the identified peptides is not currently sufficient for use in the recovery of REE from mining waters. For such an application, however, not only the affinity but in particular the selectivity is crucial. Furthermore, a high affinity is not necessarily an advantage for the regeneration of the peptides. The extent to which the peptides are suitable for real applications or how they can be improved will be the subject of future studies. Goal for our following research is the investigation of the identified peptides for their selectivity for Eu^3+^ in contrast to other Ln. Those peptides will be applied in biohybrid filter modules for the selective recovery of Eu^3+^ and, with the help of novel selected peptides, also of other Ln from mining wastewater.

## 5 Conclusion

In this study, we report on the application of various independent biopanning experiments using a commercial phage display peptide library and different elution methods in combination with NGS as a powerful sequencing method for the selection and identification of Eu^3+^ ion binding peptides. Sequences from different fractions were compared and related to each other and corresponding enrichment factors were calculated. From a large number of enriched peptide sequences after NGS, nine were finally selected and synthesized as linear dodecamers. Two peptides were additionally modified by the replacement or incorporation of aspartic or glutamic acid. Initial binding studies showed Eu^3+^ ion binding affinity for eight of the 12 peptides tested, with peptide GC9 (EALTVNIKREME) performing best of the originally selected sequences. When looking at the reverse GC9 sequence (EMERKINVTLAE), similarities can be found in the amino acid composition to the reference peptide CaM-EF4 (DIDGDGQVNYEE). An improvement in binding was observed for three of the four modified variants of the peptides GC1 (VHHVAGNALQPF) and GC3 (SIHSVTKGRYPV), but not for GC3-V5/D, which showed no complex formation. This and the lack of Eu^3+^ affinity of RB-Ar-8 (SIHSVTKGWYPV) compared to GC3 (which differs only by one amino acid at position 9) suggests the importance of certain amino acids at specific positions (V5, R9) within the peptide sequence of GC3.

With the help of PSD, we have succeeded in identifying good Eu^3+^ binders (GC9, GC1+D+E and GC3+D+E). Although these peptides do not yet bind as well as a biomolecule known from nature (CaM-EF4), our results demonstrate the power of PSD technology. The extent to which these affine peptides are suitable for the selective recovery of Eu^3+^ from e-waste, mining and industrial wastewater and possibly for the detoxification of the incorporated metal from the human body will be investigated and reported in future studies. This involves testing the peptide immobilized on a matrix under various conditions and for selectivity. Future work will focus even more on the development of selective peptides, which–in contrast to the proteins known from nature–distinguish between the rare earth metals and can only bind specific Ln^3+^ ions.

## Data Availability

The datasets presented in this study can be found in online repositories. The names of the repository/repositories and accession number(s) can be found below: https://doi.org/10.14278/rodare.3183, 10.14278/rodare.3183.
