# Supplementary material for: Application of phage surface display for the identification of Eu3+-binding peptides
Source: Front Bioeng Biotechnol. 2025 Feb 20;13:1508018. doi: 10.3389/fbioe.2025.1508018 (PMC11882594; doi:10.3389/fbioe.2025.1508018)
Supplement: Supplementary file 1 [file DataSheet1.docx]

# **Supplementary Material**


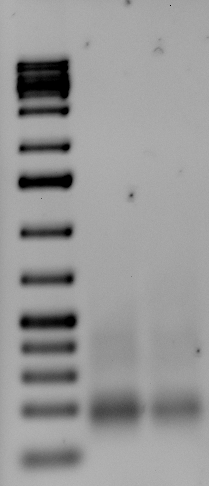


2000 bp

1500 bp

1000 bp

700 bp

500 bp

400 bp

300 bp

200 bp

75 bp

**Figure S1:** **PCR product fragments of the phage elution fractions, which were cut out of a 2 % agarose gel, purified and analyzed via NGS. This is only an example image. Not all elution fractions analyzed by NGS are shown here. DNA ladder: Thermo Scientific GeneRuler 1 kb Plus.**

**Table S1: Pipetting scheme for TRLFS titration experiments in which peptide was titrated to EuCl3 in 20 single steps.**

| Sample | Volume of EuCl_3_ in cuvette (µl) | Added volume of EuCl_3_  (µl) | Added volume of peptide  (µl) | Concentration of peptide stock solution  (µM) | Final concentration of peptide in cuvette  (µM) |
| --- | --- | --- | --- | --- | --- |
| 01 | 500 | 0 | 0.0 | 2500 | 0.0 |
| 02 | 500 | 0 | 1.0 | 2500 | 5.0 |
| 03 | 500 | 0 | 1.2 | 2500 | 11.0 |
| 04 | 500 | 0 | 2.4 | 2500 | 23.0 |
| 05 | 500 | 0 | 3.7 | 2500 | 41.0 |
| 06 | 500 | 0 | 5.0 | 2500 | 65.0 |
| 07 | 500 | 0 | 6.4 | 2500 | 95.0 |
| 08 | 500 | 0 | 7.9 | 2500 | 131.0 |
| 09 | 500 | 0 | 9.5 | 2500 | 173.0 |
| 10 | 500 | 0 | 11.3 | 2500 | 221.0 |
| 11 | 500 | 0 | 13.3 | 2500 | 275.0 |
| 12 | 500 | 0 | 15.6 | 2500 | 335.0 |
| 13 | 500 | 0 | 18.2 | 2500 | 401.0 |
| 14 | 500 | 0 | 21.2 | 2500 | 473.0 |
| 15 | 500 | 0 | 24.7 | 2500 | 551.0 |
| 16 | 500 | 0 | 28.9 | 2500 | 635.0 |
| 17 | 500 | 0 | 34.0 | 2500 | 725.0 |
| 18 | 500 | 0 | 40.3 | 2500 | 821.0 |
| 19 | 500 | 0 | 48.2 | 2500 | 923.0 |
| 20 | 500 | 0 | 58.3 | 2500 | 1031.0 |

#
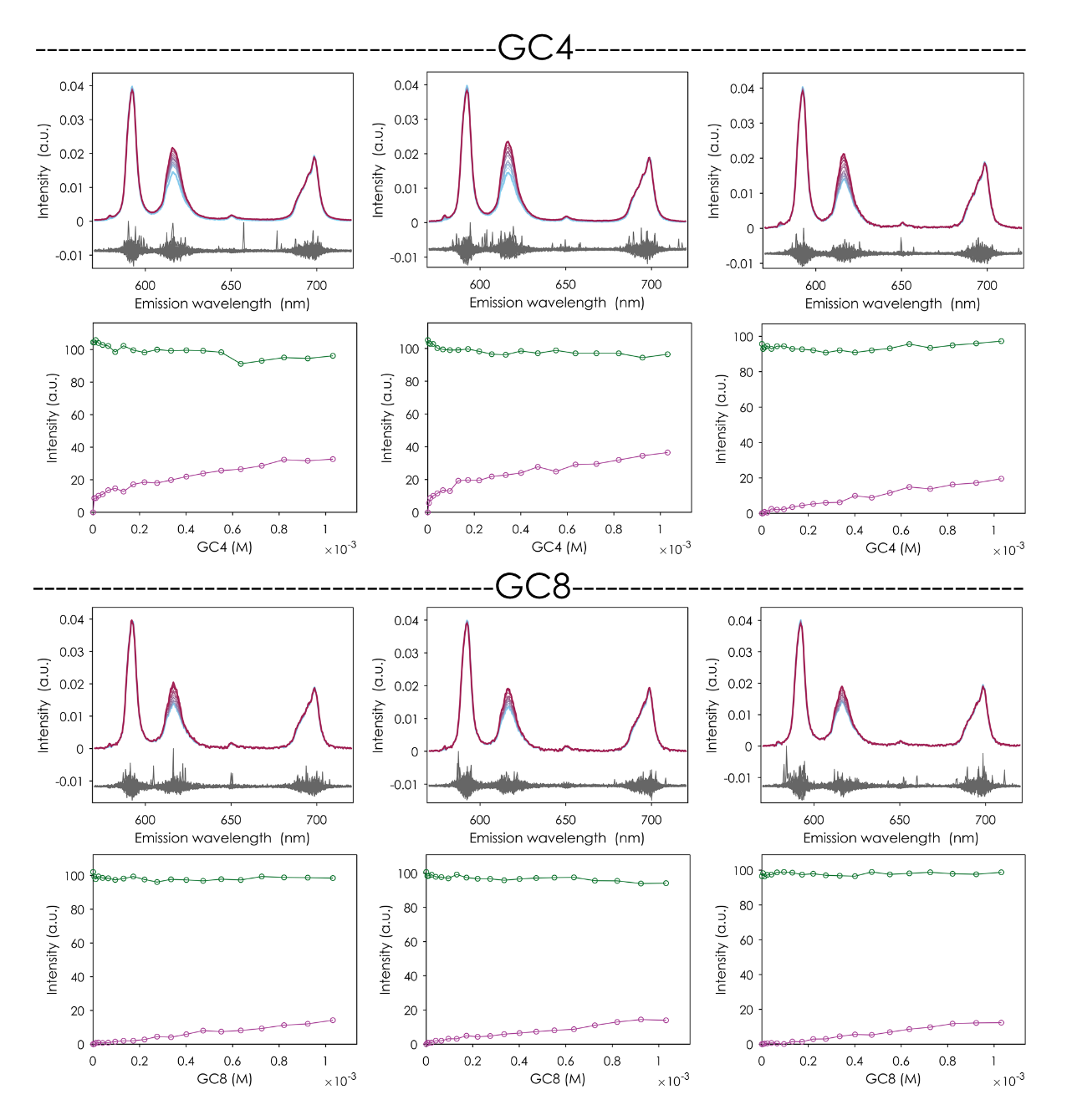


**Figure S2: Eu^3+^ complexation studies of the selected peptides with TRLFS. t_0_ fluorescence emission spectra (top each) and distribution diagrams derived from them (bottom each) with Eu^3+^ aquo ion as green line and 1:1 complex as purple line. Titration of 0 to 1031 µM peptide to 10 µM EuCl_3_ in 20 steps, pH 5.2, background 100 mM KCl.**


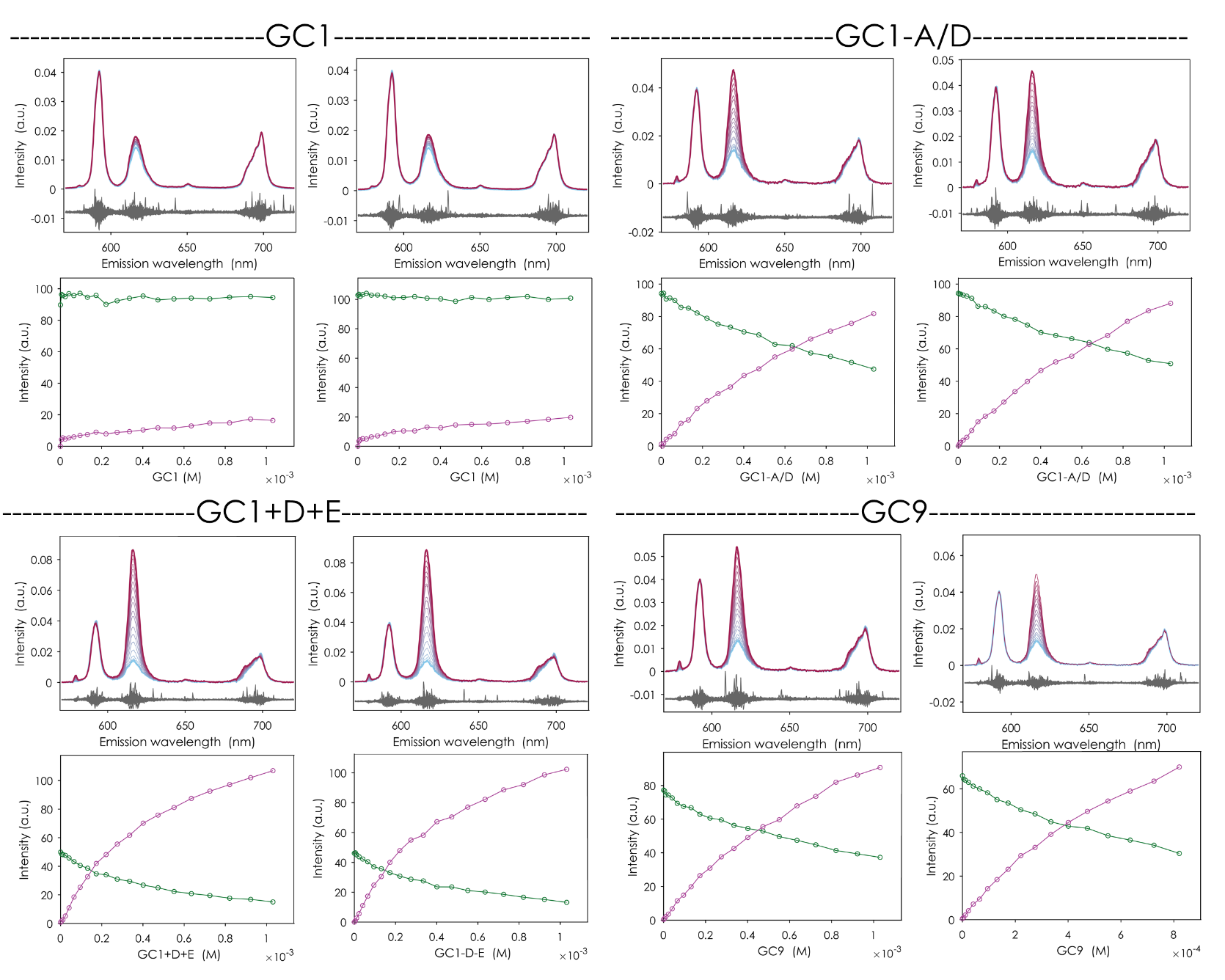


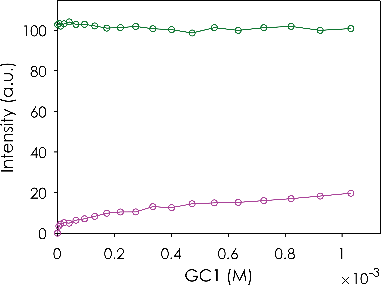

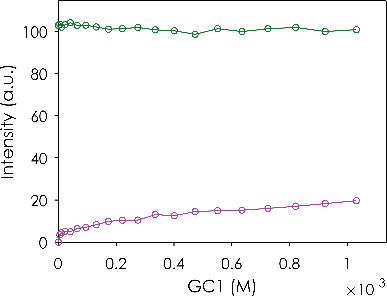


**Figure S3: Eu^3+^ complexation studies of the selected peptides with TRLFS. t_0_ fluorescence emission spectra (top each) and distribution diagrams derived from them (bottom each) with Eu^3+^ aquo ion as green line and 1:1 complex as purple line. Titration of 0 to 1031 µM peptide to 10 µM EuCl_3_ in 20 steps, pH 5.2, background 100 mM KCl.**


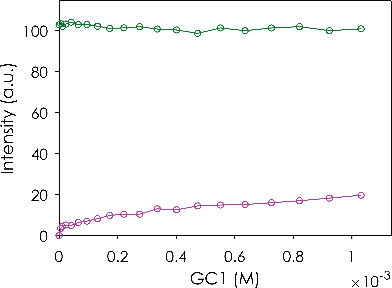

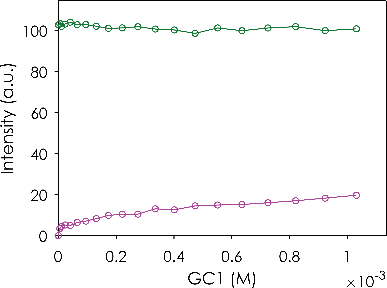


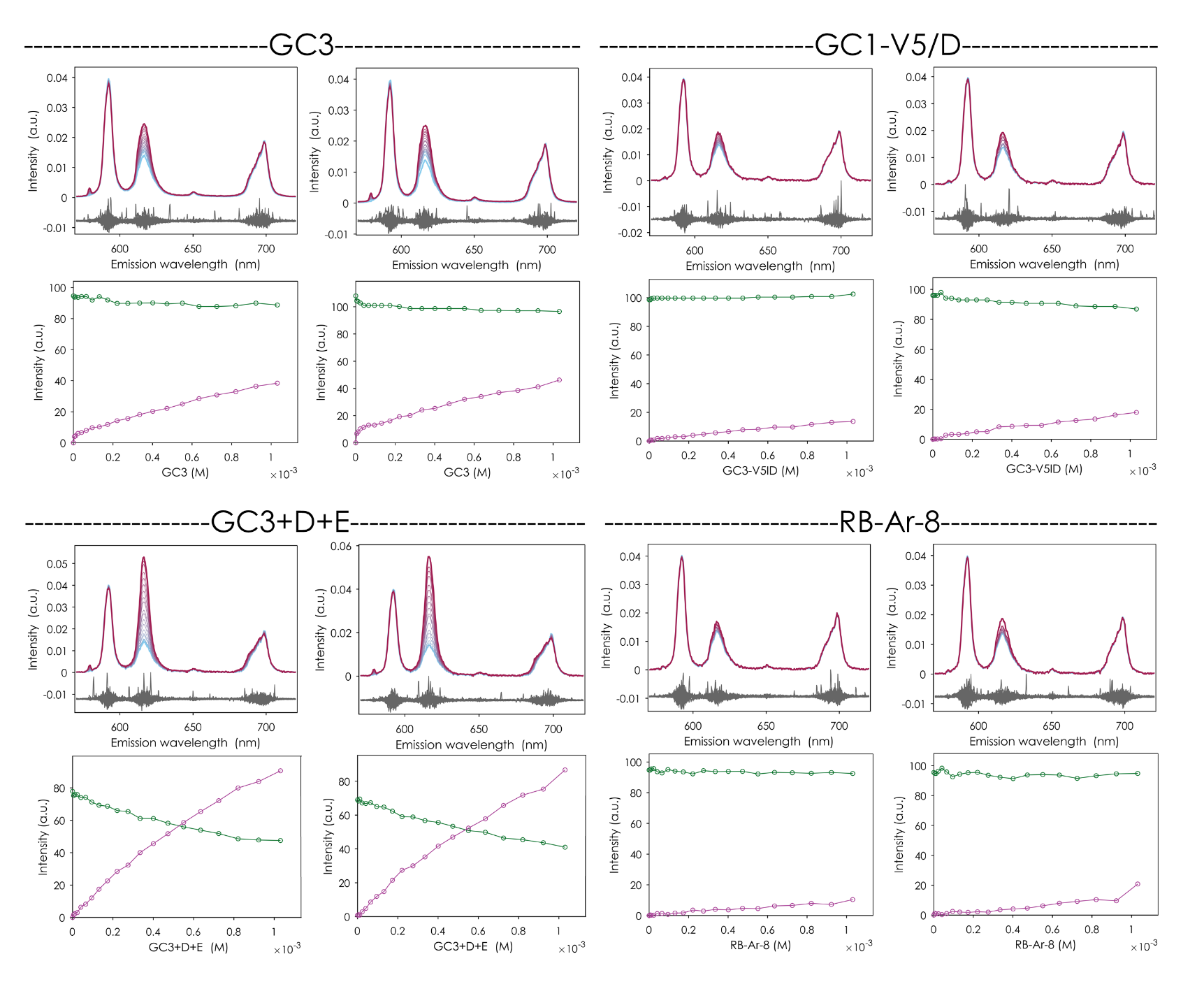


**Figure S4: Eu^3+^ complexation studies of the selected peptides with TRLFS. t_0_ fluorescence emission spectra (top each) and distribution diagrams derived from them (bottom each) with Eu^3+^ aquo ion as green line and 1:1 complex as purple line. Titration of 0 to 1031 µM peptide to 10 µM EuCl_3_ in 20 steps, pH 5.2, background 100 mM KCl.**

**Table S2: K_D_ values for the respective peptide-Eu^3+^ complexes and corresponding standard deviations from TRLFS-titration experiment. N.a. is not available. This is due to the low affinity of the corresponding peptide. The K_D_ values are then not reliable and show large fluctuations within the triplicate.**

| Peptide | K_D_ (M) | | | | Standard deviation (M) | | | |
| --- | --- | --- | --- | --- | --- | --- | --- | --- |
|  | **Titration series 1** | **Titration series 2** | **Titration series 3** | **Average** | **Titration series 1** | **Titration series 2** | **Titration series 3** | **Average** |
| GC1 | n.a. | 1.26 × 10^-1^ | 6.80 × 10^-1^ | 4.03 × 10^-1^ | n.a. | 1.95 × 10^-1^ | 3.73 × 10^-1^ | 2.84 × 10^-1^ |
| GC1-A/D | 9.38 × 10^-4^ | 1.15 × 10^-3^ | 1.25 × 10^-3^ | 1.11 × 10^-3^ | 2.65 × 10^-5^ | 2.39 × 10^-5^ | 2.61 × 10^-5^ | 2.55 × 10^-5^ |
| GC1+D+E | 4.74 × 10^-4^ | 5.11 × 10^-4^ | 4.82 × 10^-4^ | 4.89 × 10^-4^ | 1.52 × 10^-5^ | 9.68 × 10^-6^ | 8.17 × 10^-6^ | 1.10 × 10^-5^ |
| GC3 | 1.47 × 10^-2^ | n.a. | n.a. | 1.47 × 10^-2^ | 2.55 × 10^-3^ | n.a. | n.a. | 2.55 × 10^-3^ |
| GC3-V5/D | n.a. | n.a. | n.a. | n.a. | n.a. | n.a. | n.a. | n.a. |
| GC3+D+E | 1.56 × 10^-3^ | 1.46 × 10^-3^ | 1.61 × 10^-3^ | 1.54 × 10^-3^ | 3.79 × 10^-5^ | 6.43 × 10^-5^ | 4.68 × 10^-5^ | 4.97 × 10^-5^ |
| GC4 | n.a. | n.a. | n.a. | n.a. | n.a. | n.a. | n.a. | n.a. |
| GC8 | n.a. | n.a. | n.a. | n.a. | n.a. | n.a. | n.a. | n.a. |
| GC9 | 1.30 × 10^-3^ | 1.05 × 10^-3^ | 7.98 × 10^-4^ | 1.05 × 10^-3^ | 3.33 × 10^-5^ | 2.59 × 10^-5^ | 1.5 × 10^-5^ | 2.47 × 10^-5^ |

**
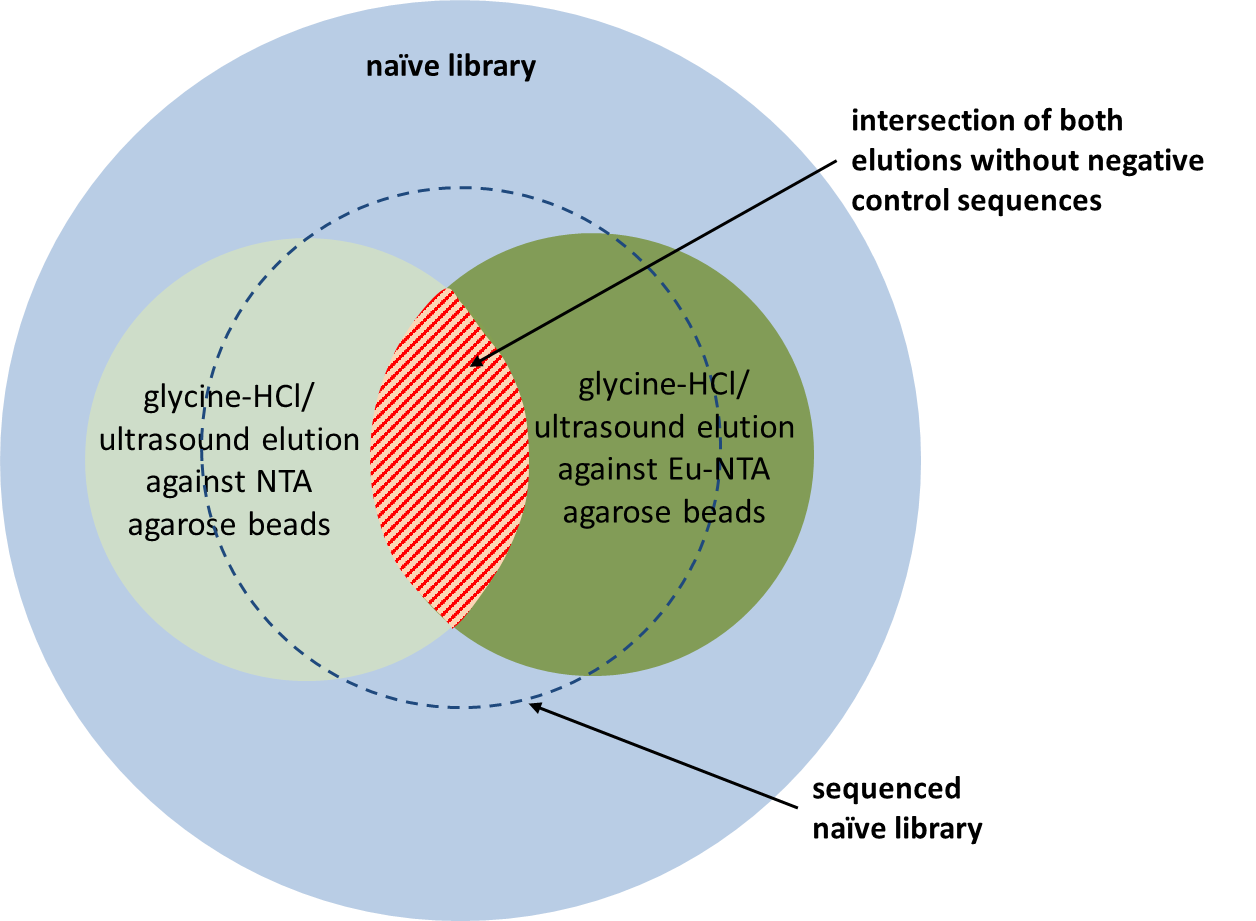
**

**Figure S5:** **Venn diagram of the sequences of different elution fractions, visualizing experiment 2.6.2.**

**
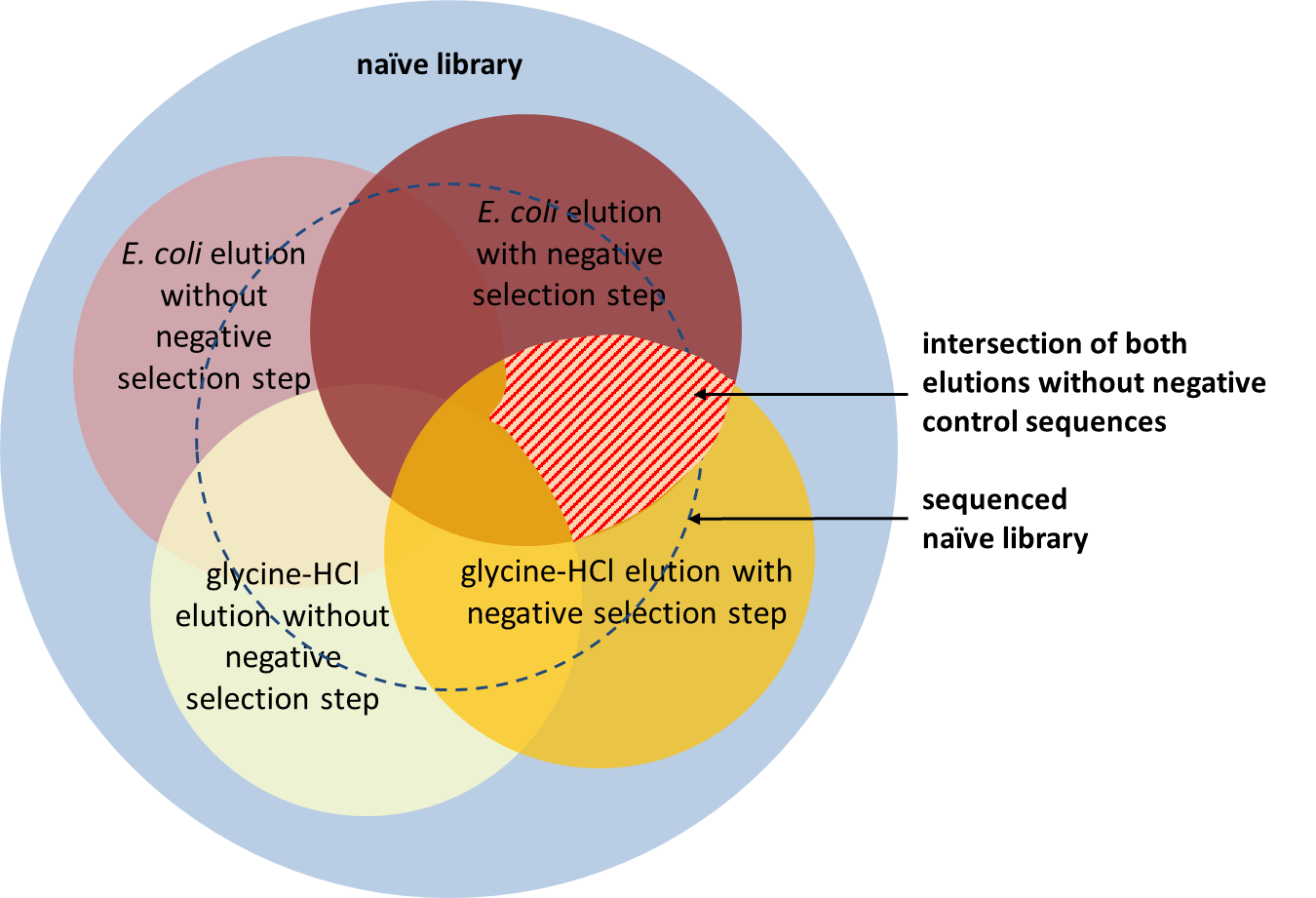
**

**Figure S6: Venn diagram of the sequences of different elution fractions, visualizing experiment 2.6.3.**

**Table S3: Sanger sequenced peptide sequences of the 12mer peptides of 107 selected phage clones from the E. coli elution of the first panning strategy 2.6.1 and their frequencies and amino acid properties (1).**

| Sequence motif | Frequency | | Amino acid properties | | | |
| --- | --- | --- | --- | --- | --- | --- |
|  | **absolute** | **relative [%]** | **hydrophobic** | **hydrophilic (polar uncharged)** | **basic (positive charged)** | **acidic (negative charged)** |
| MKAHHSQLYPRH | 48 | 44,9 | 4 | 3 | 5 | 0 |
| VPRHSHPLINMR | 24 | 22,4 | 6 | 2 | 4 | 0 |
| LTPHKHHKHLHA | 3 | 2,8 | 4 | 1 | 7 | 0 |
| GVRPVHLHKTHS | 2 | 1,9 | 4 | 3 | 5 | 0 |
| SPNHLHHWKHPA | 2 | 1,9 | 5 | 2 | 5 | 0 |
| NATFAHHKPSKW | 2 | 1,9 | 5 | 3 | 4 | 0 |
| FTPSDLARPTSL | 1 | 0,9 | 6 | 4 | 1 | 1 |
| ISPHSHSLLKHS | 1 | 0,9 | 4 | 4 | 4 | 0 |
| WPKNGDHRHNTP | 1 | 0,9 | 3 | 4 | 4 | 1 |
| HGRMDPRGVYTA | 1 | 0,9 | 4 | 4 | 3 | 1 |
| NLTTWHSSQARS | 1 | 0,9 | 3 | 7 | 2 | 0 |
| TPHGYQPMQGKT | 1 | 0,9 | 3 | 7 | 2 | 0 |
| STMPHKPYQRGP | 1 | 0,9 | 4 | 5 | 3 | 0 |
| QLKNVNNHFTKH | 1 | 0,9 | 3 | 5 | 4 | 0 |
| IQQHDVPGLPNN | 1 | 0,9 | 5 | 5 | 1 | 1 |
| HKAHPPTNYTNS | 1 | 0,9 | 3 | 6 | 3 | 0 |
| KPVYHHLKPYLT | 1 | 0,9 | 5 | 3 | 4 | 0 |
| GLTFQVPWHANM | 1 | 0,9 | 7 | 4 | 1 | 0 |
| FQARWEPPRLLQ | 1 | 0,9 | 7 | 2 | 2 | 1 |
| GLQNVHHTKLPT | 1 | 0,9 | 4 | 5 | 3 | 0 |
| KMPHKWYMDNSA | 1 | 0,9 | 5 | 3 | 3 | 1 |
| ARSLEPAPSRHS | 1 | 0,9 | 5 | 3 | 3 | 1 |
| GCNHDSCSALTK | 1 | 0,9 | 2 | 7 | 2 | 1 |
| EALTVNIKREME | 1 | 0,9 | 5 | 2 | 2 | 3 |
| KTMFPYHPWHHL | 1 | 0,9 | 6 | 2 | 4 | 0 |
| KMKIPWYELQSS | 1 | 0,9 | 5 | 4 | 2 | 1 |
| VPHKNILVSLHH | 1 | 0,9 | 6 | 2 | 4 | 0 |
| YDHTHKQPWHLH | 1 | 0,9 | 3 | 3 | 5 | 1 |
| SHDTRSPFTWGR | 1 | 0,9 | 3 | 5 | 3 | 1 |
| YPSAHHSLMRPA | 1 | 0,9 | 6 | 3 | 3 | 0 |
| KLYHKPLDGHLA | 1 | 0,9 | 5 | 2 | 4 | 1 |
| HNAKHTFTAHSL | 1 | 0,9 | 4 | 4 | 4 | 0 |

**Table S4: Sanger sequenced peptide sequences of the 12mer peptides of 38 selected phage clones from the glycine-HCl elution (0.2 M) of the first panning strategy 2.6.1 and their frequencies and amino acid properties (1).**

| Sequence motif | Frequency | | Amino acid properties | | | |
| --- | --- | --- | --- | --- | --- | --- |
|  | **absolute** | **relative [%]** | **hydrophobic** | **hydrophilic (polar uncharged)** | **basic (positive charged)** | **acidic (negative charged)** |
| GNWHARPGDHHR | 19 | 50,0 | 3 | 3 | 5 | 1 |
| YGWPHTSRLHSA | 11 | 28,9 | 4 | 5 | 3 | 0 |
| YTPNAASHNNLR | 2 | 5,3 | 4 | 6 | 2 | 0 |
| LTPHKHHKHLHA | 2 | 5,3 | 4 | 1 | 7 | 0 |
| GTHHAWANKSAR | 1 | 2,6 | 4 | 4 | 4 | 0 |
| NAWKQKDHVLQV | 1 | 2,6 | 5 | 3 | 3 | 1 |
| GPTADHFHNRHE | 1 | 2,6 | 3 | 3 | 4 | 2 |
| MKAHHSQLYPRH | 1 | 2,6 | 4 | 3 | 5 | 0 |

**REFERENCES**

1. Claus G. *Selektion hochaffiner Peptide zur Bindung von f-Elementen und deren physikochemische Charakterisierung*. Master thesis. Zittau (2020).
